# Global Transcriptome Analyses Reveal Differentially Expressed Genes of Six Organs and Putative Genes Involved in (Iso)flavonoid Biosynthesis in *Belamcanda chinensis*

**DOI:** 10.3389/fpls.2018.01160

**Published:** 2018-08-14

**Authors:** Mei Tian, Xiang Zhang, Yan Zhu, Guoyong Xie, Minjian Qin

**Affiliations:** ^1^Department of Resources Science of Traditional Chinese Medicines, School of Traditional Chinese Pharmacy, China Pharmaceutical University, Nanjing, China; ^2^Key Laboratory of Modern Traditional Chinese Medicines (Ministry of Education), China Pharmaceutical University, Nanjing, China

**Keywords:** *Belamcanda chinensis*, RNA-sequencing, *de novo* assembly, differentially expressed gene, flavonoid biosynthesis, isoflavonoid biosynthesis, CYP 450s, transcription factors

## Abstract

*Belamcanda chinensis* (L.) DC., a perennial herb of the family Iridaceae, is rich in a variety of (iso)flavonoids with significant organ-specific distribution and has a swollen rhizome that is widely used in East Asia as a traditional medicine. In the present study, comprehensive transcriptomes of six organs (root, rhizome, aerial stem, leaf, flower, and young fruit) of *B. chinensis* were obtained by high-throughput RNA-sequencing and *de novo* assembly. A total of 423,661 unigenes (mean length = 618 bp, median length = 391 bp) were assembled and annotated in seven databases: Non-redundant protein sequences, Nucleotide sequences, Swiss-Prot, Protein family database, euKaryotic Ortholog Groups, Kyoto Encyclopedia of Genes and Genomes (KEGG), and Gene Ontology (GO). A total of 4995 transcription factors were identified, including 408 MYB, 182 bHLH, 277 AP2/ERF, and 228 WRKY genes. A total of 129 cytochrome P450 unigenes belonging to 10 divergent clans were identified and grouped into clades in a phylogenetic tree that showed their inferred evolutionary relationship. Differentially expressed unigenes among the six organs were subjected to GO and KEGG enrichment analysis to profile the functions of each organ. Unigenes associated with (iso)flavonoid biosynthesis were then profiled by expression level analysis. Additionally, the complete coding sequences of six predicted enzymes essential to the (iso)flavonoid pathway were obtained, based on the annotated unigenes. This work reveals clear differences in expression patterns of genes among the six organs and will provide a sound platform to understand the (iso)flavonoid pathways in *B. chinensis.*

## Introduction

*Belamcanda chinensis* (L.) DC. [syn. *Iris domestica* (L.) Goldblatt et Mabb], known as “Shegan” in Chinese, is a traditional medicinal herb whose rhizome has been used as a traditional medicine for treating coughing, asthma, pharyngitis, and many other throat troubles since the Han Dynasty, 2000 years ago. It was also adopted as one Chinese medicinal herb in the European Pharmacopeia (9th edition). The rhizome of *B. chinensis* is rich in a variety of phenolic compounds, especially flavonoids and isoflavonoids. Isoflavones such as tectoridin, iridin, tectorigenin, irigenin and irisflorentin are demonstrated as the major bioactive constituents in the rhizome. These show good pharmacological activities as antioxidants, selective estrogen receptor modulators, and anti-proliferative agents, and have the prospect of preventing fatal diseases such as cancer, atherosclerosis, osteoporosis, hyperlipidemia, cardiovascular diseases, and ischemic stroke (Jung et al., 2003; Morrissey et al., 2004; Seidlová-Wuttke et al., 2004; Chen et al., 2011; Wu et al., 2011; Liu et al., 2012; Li et al., 2016c). The major class of *Belamcanda*-isoflavones is represented with the distinctive 6-methoxylated structure. The 6-methoxylation and 5-hydroxylation can increase the potency of inhibition of PGE2 production, and 7-*O-*glycosylation decreases this inhibitory activity (Kim et al., 1999; Shin et al., 1999; Yamaki et al., 2002).

Isoflavonoids are a predominantly legume-specific subclass of flavonoid metabolites, with important roles in defense and nodulation in some leguminous plants (Subramanian et al., 2006, 2007; Algar et al., 2014). Isoflavonoids have also been found in some non-leguminous plants belonging to Iridaceae, Moraceae, Podocarpaceae, and Rosaceae (Lapcik, 2007). Although the mechanism of isoflavonoid biosynthesis in legumes has been studied extensively, it remains scant for non-leguminous plants. In legumes, isoflavonoids are produced via a branch of the phenylpropanoid pathway. The first three enzymes in phenylpropanoid biosynthesis are phenylalanine ammonia lyase (PAL), cinnamate 4-hydroxylase (C4H), and *p*-coumaroyl coenzyme A ligase (4CL), which produce *p*-coumaroyl-CoA as a precursor for the synthesis of chalcone. Via a condensation reaction with three molecules of malonyl CoA, *p*-coumaroyl-CoA can be catalyzed to naringenin chalcone by chalcone synthase (CHS), the first key enzyme in flavonoid biosynthesis. Next, chalcone isomerase (CHI) catalyzes the chalcones, producing flavanones, which are the substrates for many downstream enzymatic reactions to form flavonoids. Isoflavonoids are derived from the flavonoid biosynthesis pathway via liquiritigenin or naringenin in legume plants. The key step at the very beginning of the isoflavonoid metabolic pathway is the oxidation of flavanone with the migration of an aryl moiety from C2 to C3 to form a 2-hydroxyisoflavanone mediated by isoflavone synthase (IFS), which has been identified in multiple legume plants and sugar beet (Chenopodiaceae) (Jung et al., 2000). The 2-hydroxyisoflavanones are then dehydrated by 2-hydroxyisoflavanone dehydratase (HID) (Shimamura et al., 2007) to produce isoflavones, which act as substrates of various isoflavone-specific hydroxylases, *O-*methyltransferases, and *O-*glucosyltransferases.

As most medicinal plants are non-model organisms with little available genomic information, the mechanisms of regulation of development, biosynthesis of metabolites, and other physiological process remain little understood. Fortunately, with the remarkable development of next-generation sequencing technologies (Metzker, 2010) and assembly software, deep sequencing of the transcriptome (also known as RNA-Seq) can provide both the sequence and frequency of transcripts present at any particular time in a specific cell, tissue, or organ. The annotation and expression analysis of transcripts can be applied for the identification of candidate genes participating in many physiological processes. RNA-Seq is increasingly applied to study a variety of medicinal plants and has proved particularly efficient in non-model species (Chen et al., 2015). Although *B. chinensis* is an important medicinal plant, its genomic information is almost unknown—neither RNA Seq nor expressed sequence tags have been reported. This seriously restricts study of the biosynthesis pathways of its secondary metabolites. As a non-leguminous plant with the most abundant isoflavonoids, *B. chinensis* is ideal to explore the isoflavonoid pathway in non-leguminous plants. Hence, the high-throughput transcriptome sequencing of *B. chinensis* is indispensable to discover genes associated with isoflavonoid biosynthesis. In the present work, comprehensive transcriptome and isoflavonoid profile analyses on six organs (the root, rhizome, aerial stem, leaf, flower, and young fruit) of *B. chinensis* were performed to identify the genes essential for the biosynthesis of (iso)flavonoids, as well as to profile the expression patterns of different organs at great length. Additionally, we cloned six predicted key enzyme genes in the upstream of (iso)flavonoid biosynthesis pathway of *B. chinensis* based on annotated unigenes. The present work will provide insight into the biosynthesis, and accumulation of (iso)flavonoids in *B. chinensis.*

## Materials and Methods

### Plant Material

The seeds of *B. chinensis* were collected from Anguo, Hebei province, China. After germinating in the clean sands, the 14-day-old well-growing seedings were planted in polyethylene plastic disks filled with nutrient soil. The plants of *B. chinensis* were grown for 3 years under natural day/night conditions in the greenhouse located in the Botanic Garden of China Pharmaceutical University, Nanjing, China (31°54′36.36 N, 118°54′38.42 E) with uniform management, including regular watering, weeding, and fertilization. Fresh samples of six organs (root, rhizome, aerial stem, leaf, flower, and young fruit) (**Supplementary Figure 1**) were collected separately in three biological replicates from three independent plants of *B. chinensis* during their flowering–fruiting period in early July. The samples were washed clean, frozen immediately in liquid nitrogen, and then kept at -80°C until extraction of chemical constituents and RNA.

**FIGURE 1:**
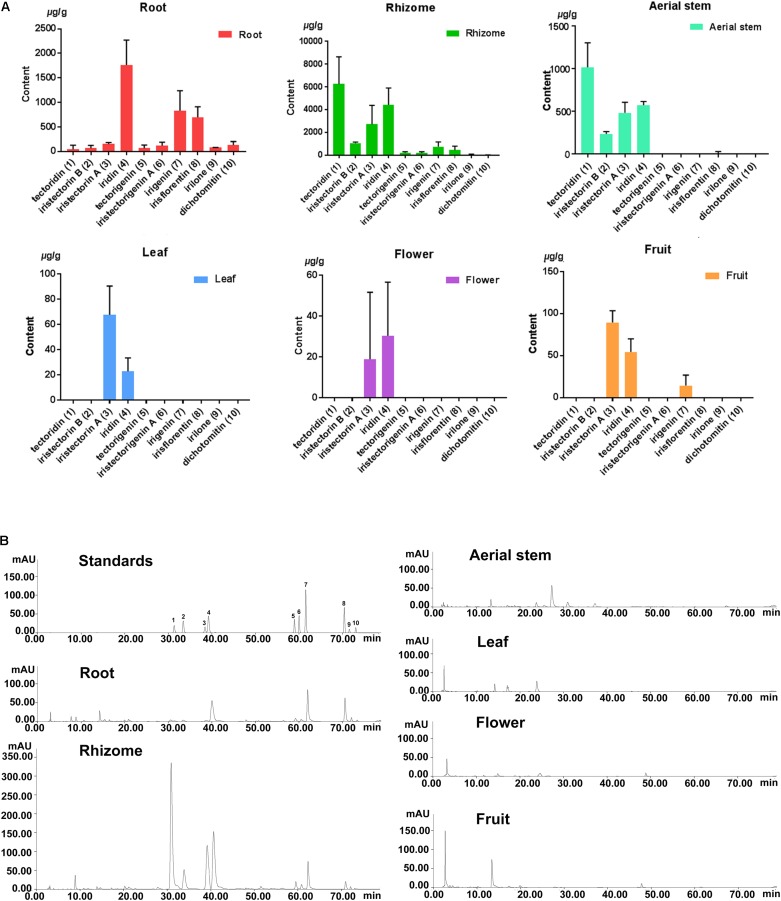
**(A)** Contents of 10 isoflavonoids in the six organs of *B. chinensis.* **(B)** HPLCs of extracts obtained from the organs of *B. chinensis* at 269 nm wavelength. (1) Tectoridin, (2) Iristectorin A, (3) Iristectorin B, (4) Iridin, (5) Tectorigenin, (6) Iristectorigenin A, (7) Irigenin, (8) Irisflorentin, (9) Irilone, (10) Dichotomitin.

### Quantitative Analysis of Isoflavonoids in *B. chinensis*

About 0.5 g of each sample was ground into powder and twice extracted with 3 mL of 75% methanol in an ultrasonic bath for 15 min. Slurry of each sample was finally made up to 10 mL. The supernatant was obtained by centrifugation (13,000 × *g*) prior to use. Each standard compound was accurately weighed, separately dissolved, diluted, and mixed to obtain a mixed standard stock solution at a certain concentration with 75% methanol. The chromatographic conditions were as outlined by Zhu et al. (2014).

### RNA Extraction

Total RNA from the above samples was isolated with an RNAsimple total RNA kit (TIANGEN, Beijing, China). RNA purity and concentration were checked using a NanoPhotometer spectrophotometer (IMPLEN, Westlake Village, CA, United States) and Qubit RNA Assay Kit in Qubit 2.0 Fluorometer (Life Technologies, Carlsbad, CA, United States). RNA integrity was assessed using the RNA Nano 6000 Assay Kit of the Agilent Bioanalyzer 2100 system (Agilent Technologies, Santa Clara, CA, United States). High-quality total RNA (RNA integrity number > 7.4) was used for transcriptome sequencing by Illumina platform.

### Transcriptome Sequencing, Data Filtering, and *de novo* Assembly

There were 18 sequencing libraries, respectively, generated from 1.5 μg of total RNA per sample using a NEB Next Ultra RNA Library Prep Kit for Illumina (NEB, Seattle, WA, United States) and subjected to sequencing on the Illumina HiSeq 2500 platform (Illumina, San Diego, CA, United States) in paired-end 125 base runs, by following standard protocols. Clean data were obtained, by removing low-quality reads and reads containing adapters and poly-*N* from the raw data, and assembled into contigs using the Trinity assembler (r20140413p1) with min_kmer_cov set to 1 and other parameters set by default. This system has shown good performance in assembly of low-expression genes with deep transcriptome coverage in species without a reference genome (Grabherr et al., 2011). The longest transcripts were considered as unigenes for functional annotation by identifying nucleotide sequence of all transcripts.

### Functional Annotation of Unigenes

Unigenes were annotated based on NCBI non-redundant protein sequences (Nr), NCBI non-redundant nucleotide sequences (Nt), Swiss-Prot, and euKaryotic Ortholog Groups/Clusters of Orthologous Groups of proteins (KOG/COG^1^) using NCBI BLAST 2.2.28+ (Nr, Nt, and Swiss-Prot: *e*-value = 1*e-*5, KOG/COG: *e*-value = 1*e-*3). All unigenes were searched against the Protein family (Pfam) database by hmmscan (HMMER 3, *e*-value = 0.01) (Punta et al., 2012). GO annotations of the unigenes were obtained using the BLAST2 GO program (b2g4pipe_v2.5, *e*-value = 1*e-*6) (Conesa and Gotz, 2008) based on the annotation of Nr and Pfam. Kyoto Encyclopedia of Genes and Genomes (KEGG) (Kanehisa et al., 2008) is a database resource for determining high-level functions and utilities of biological systems from molecular-level information^2^. Unigenes were assigned to KEGG pathways by the online KEGG Automatic Annotation Server (KAAS, r140224^3^, *e*-value = 1*e-*10) (Moriya et al., 2007) and mapped to plant-specific pathways according to KEGG Plant Resource (Kanehisa, 2016). Transcription factors (TFs) were predicted using iTAK^4^ (Zheng et al., 2016).

### Quantification of Gene Expression Levels and Differential Expression Analysis of the Organs

Clean data of each sample were mapped back onto the assembled sequences and the read count for each transcript was obtained from the mapping results using the RNA-Seq by Expectation–Maximization (RSEM, v1.2.15) software package (Li and Dewey, 2011) for estimating gene expression levels. The read counts were normalized by the edgeR program package (Robinson et al., 2010) through one scaling normalized factor for each sequenced library. The FPKM (fragments per kilobase of exon model per million mapped reads) was calculated and used to quantify expression abundance of transcripts in each sample. Differential expression analysis of the six organs was performed with the DESeq R package (1.10.1) (Anders and Huber, 2010). A model based on the negative binomial distribution was applied for determining differential expression in digital gene expression data. The *P*-value was adjusted using the Benjamini and Yekutieli’s (2001) approach to control the false discovery rate. Genes with an adjusted *P*-value < 0.005 and |log_2_ Fold_change| > 1 were assigned as differentially expressed. All Venn diagrams were created by a convenient and powerful online tool^5^ (Heberle et al., 2015).

### GO and KEGG Pathway Enrichment Analysis of Differentially Expressed Genes (DEGs)

The GO enrichment of DEGs was performed with the GO seq R package based on the Wallenius non-central hyper-geometric distribution (Young et al., 2010), which can adjust for gene length bias in DEGs. KOBAS was used (Mao et al., 2005) to test the statistical enrichment of differential expression genes in KEGG pathways.

### Identification and Classification of Cytochrome P450 (CYP450) Proteins

Deduced proteins based on the predicted open-reading frames of the unigenes were subjected to BLASTP analysis via NCBI and Cytochrome P450 database^6^. The putative CYP450 proteins were named based on the homology to the reference plant CYP450 proteins and further manually confirmed using the ExPASy-PROSITE tool (de Castro et al., 2006)^7^. To analyze the phylogenetic relationship, the CYP450 proteins were aligned with the UPGMB clustering method (gap opening –2.9, gap extension penalty 0, and hydrophobicity 1.2) in the MUSCLE module (Edgar, 2004) in MEGA 7 (Kumar et al., 2016). A phylogenetic tree was constructed using the neighbor-joining method (Saitou and Nei, 1987) with bootstrap testing using 1000 replications (Felsenstein, 1985) and evolutionary distances computed using Poisson correction (Zuckerkandl and Pauling, 1965).

### Validation of Gene Expression Level With Real-Time PCR

Fifteen pairs of primers of selected unigenes from RNA-Seq data were designed using Primer Express (v3.0) software (Applied Biosystems, Foster City, CA, United States) (**Supplementary Table 1**). Unigene c61429_g32, predicted to be an *Actin* gene showing relatively constant levels of FPKM in the six organs, was chosen as an internal control gene for normalization. Three biological replications were used for cDNA synthesis with a Prime Script RT reagent kit (TaKaRa, Beijing, China) following the manufacturer’s instructions. The reaction was carried out in a total volume of 20 μL with SYBR Premix Ex Taq II (TliR Nase H Plus) kit (TaKaRa, Beijing, China) using an ABI StepOne plus Real-Time PCR system (Applied Biosystems, Foster City, CA, United States), with cycling parameters of 95°C for 30 s, 40 cycles of 95°C for 5 s, and 60°C for 30 s. The double delta Ct method was used to calculate the expression level of each unigene (Livak and Schmittgen, 2001). The specificity of primers was checked by plotting a melting curve and amplification efficiency was calculated using the slope of the standard curve. For RNA-Seq analysis, the log_2_-value using the average of mean count values from three biological replicates was calculated and used as fold change in expression. The expression levels of each detected unigene in different organs were normalized to the root group.

**Table 1:** Overview of DEGs of the 15 comparisons.

Organ	DEGs vs. root	DEGs vs. rhizome	DEGs vs. aerial stem	DEGs vs. leaf	DEGs vs. flower
Rhizome	4851				
Aerial stem	3214	564			
Leaf	12,659	11,629	2337		
Flower	6907	12,321	5094	14,474	
Fruit	12,236	5442	1079	4158	3762


### Cloning of Complete Coding Sequences of Predicted Enzyme Genes Involved in (Iso)Flavonoid Biosynthesis

Six pairs of primers were designed to obtain the complete coding sequences of predicted PAL, C4H, 4CL, CHS, and two CHI isoform genes based on the annotated sequences (c58616_g31, c70487_g11, c72446_g21, c69867_g26, c69867_g24, and c62980_g1) (**Supplementary Table 1**). Total RNA of the six organs was equally mixed for the synthesis of a cDNA library using an M-Mu LV First Strand cDNA Synthesis Kit (Sangon Biotech, Shanghai, China). PCRs were carried out according to the protocol of a KOD-Plus-Kit (TOYOBO, Shanghai, China). The PCR-specific products were purified and then warmed with Taq DNA polymerase reaction mixture (Sangon Biotech, Shanghai, China) at 72°C for 30 min. The fragments were then cloned into pMD 18-T vector (TaKaRa, Beijing, China) and transformed into *Escherichia coli* DH5α competent cells. The sequences were blasted in the Nr database using the BLASTX program. The parameters of each protein were predicted by online software^8,9^.

## Results

### Quantitative Analysis of Isoflavonoid Contents in Six Organs of *B. chinensis*

Tectoridin, iristectorin A, iristectorin B, iridin, tectorigenin, iristectorigenin A, irigenin, irisflorentin, irilone, and dichotomitin were the predominant isoflavonoids in *B. chinensis*. The rhizome and root contained abundant amounts of most ingredients, and aerial stem possessed low amounts of tectoridin, iristectorin B, iristectorin A, and iridin (**Figure 1**). The contents of each isoflavonoid in the flower, fruit, and leaf were near or under the quantification limit (**Supplementary Table 2**). In the rhizome, tectoridin, followed by its aglycone tectorigenin and iridin were the most abundant compounds. The root accumulated more aglycones and aerial stem contained more glycosides. Each organ accumulated quite different amount of isoflavonoids and displayed various distribution patterns.

**Table 2:** Overview of annotations of OSGs in the organs.

	Root	Rhizome	Aerial stem	Leaf	Flower	Fruit
Annotated in Nr	1336	420	739	897	1810	382
Annotated pfam	1078	363	631	774	1529	327
Annotated in KEGG	429	75	106	547	549	113
Annotated in GO	498	248	432	782	1553	328
Annotated in at least	1336	420	783	1408	1810	383
one database (percentage)	(45.4%)	(23.1%)	(22.3%)	(36.3%)	(34.5)%	(33.0%)
Number of OSGs	2945	1817	3505	3877	5246	1162


### RNA-Seq and *de novo* Transcriptome Assembly

**FIGURE 2:**
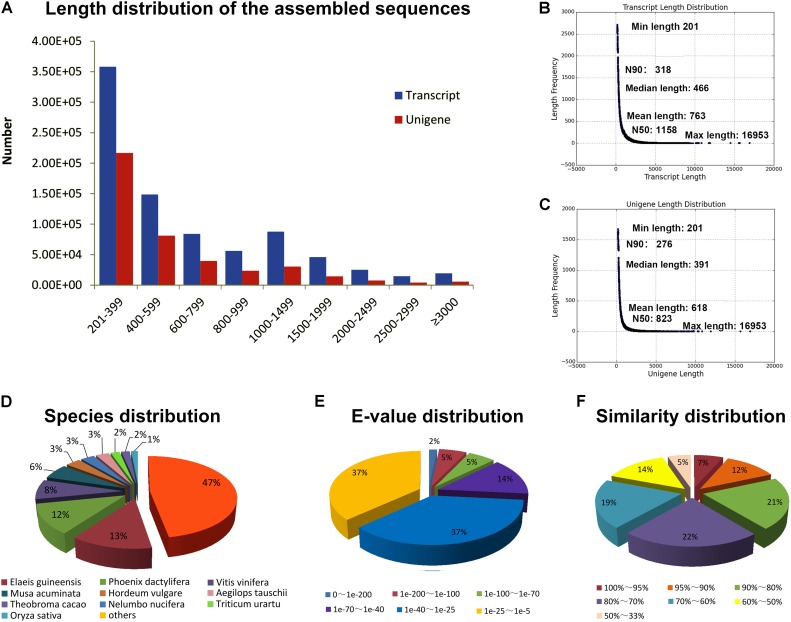
**(A)** Length distribution of the assembled sequences. **(B)** Overview of the transcript length. **(C)** Overview of the unigene length. **(D)** Species distribution of the unigenes annotated in Nr database. **(E)** *E*-value distribution of the unigenes annotated in Nr database. **(F)** Similarity distribution of the unigenes annotated in Nr database.

The transcriptomes of six organs including the root, rhizome, aerial stem, young leaf, flower, and young fruit of *B. chinensis* were separately sequenced using Illumina paired-end sequencing technology. After removing low-quality reads and reads containing adapters or poly-*N*, we obtained a total of 896,129,418 (96.59%) high-quality sequencing reads (126.64 G of clean bases) from 927,761,790 raw reads. The overview of the quality of reads is shown in **Supplementary Table 3**. A total of 840,241 transcripts were assembled with the Trinity method and 423,661 unigenes were screened from the transcripts by choosing the longest transcripts of the same genes. The length distribution of the unigenes is shown in **Figure 2A**. The length of transcripts and unigenes ranged within 201–16,953 bp. The mean length, median length, and N50 of transcripts and unigenes are shown in **Figures 2B,C**.

**Table 3:** Predicted parameters of the six cloned proteins.

	CDS length (bp)	Stop codon	G+C(%)	Length (aa)	Molecular weight of protein	Theoretical pI	Grand average of hydropathicity	Aliphatic index	Instability index	Transme-mbrane helices
BcPAL1	2163	TAG	58.6	721	78,086.88	5.79	-0.140	92.83	33.14	Outside
BcC4H1	1515	TGA	53.1	505	57,989.55	9.33	-0.039	95.19	41.53	Outside
Bc4CL1	1629	TGA	55.0	543	59,560.89	5.28	0.032	101.58	32.93	Outside
BcCHS1	1164	TAA	57.0	388	42,617.98	5.59	-0.039	95.19	41.53	Outside
BcCHI 1	630	TAA	49.1	210	23,175.51	4.85	-0.149	92.90	28.65	Outside
BcCHI 2	630	TAA	53.1	210	23,362.81	5.00	-0.056	97.90	36.82	Outside


### Functional Annotation of Unigenes

There were 134,533 (31.76%), 93,686 (22.11%), and 81,791 (19.30%) unigenes of *B. chinensis* annotated in the Nr, Swiss-Prot, and Nt databases. A total of 102,944 (24.30%) unigenes were annotated in the Pfam database via HMMER 3.0 package with *e*-value = 0.01. The annotated unigenes were compared against other plant species. Most unigenes were homologous to those of *Elaeis guineensis*, followed by *Phoenix dactylifera, Vitis vinifera*, and *Musa acuminata* (**Figure 2D**). Only 443 queries were matched to those of the plants belonging to Iridaceae. The distribution of *e*-values and similarity of annotated unigenes are shown in **Figures 2E,F**, respectively. The unigenes that did not match with any other species were considered unique to *B. chinensis*.

**FIGURE 3:**
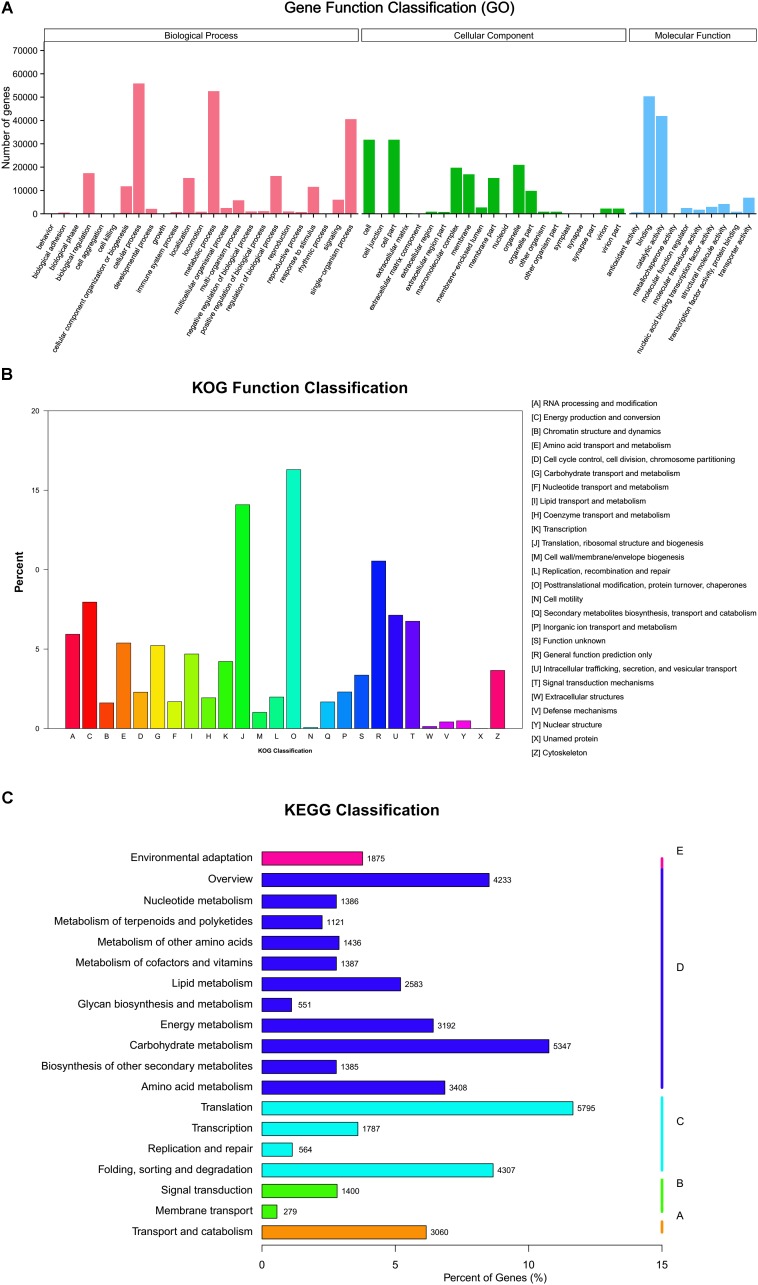
**(A)** Gene ontology function classifications of unigenes. **(B)** Clusters of orthologous function classifications of unigenes. **(C)** Kyoto Encyclopedia of Genes and Genomes classifications of unigenes mapped to 19 sub-hierarchy pathways.

A total of 104,946 (24.77%) unigenes were assigned to GO terms and divided into three ontologies: “molecular function,” “biological processes,” and “cellular component.” In the “biological process” category, most unigenes were assigned to “cellular process,” followed by “metabolic process” and “single-organism process.” The most representative function in the “molecular function” category was “binding” and “catalytic activity.” In the “cellular components” category, the major classes of genes were “cell,” “cell part,” and “organelle” (**Figure 3A**). There were 35,228 (8.32%) unigenes assigned to 26 categories by letters of the alphabet (**Figure 3B**) in the KOG database. The category of “post translational modification, protein turnover, and chaperones” (5722 unigenes) was the largest group, followed by “translation, ribosomal structure and biogenesis” (4946), “general function prediction only” (3702), and “conversion” (2794). A total of 49,747 unigenes were annotated against KEGG Orthology (KO) with Enzyme Commission (EC) numbers and assigned to 19 sub-hierarchies (**Figure 3C**), which were further divided into 131 KEGG pathways. The “translation”; “carbohydrate metabolism”; and “folding, sorting, and degradation” were the most representative pathways. More details of the GO classifications, KOG categories, and KEGG pathways are given in **Supplementary Table 4**.

**Table 4:** Chemical structures of isoflavonoids in legumes and *B. chinensis*.

		R1	R2	R3	R4	R5	R6
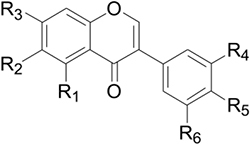	Glycitein	H	OCH_3_	OH	H	OH	H
	Genistein	OH	H	OH	H	OH	H
	Daidzein	H	H	OH	H	OH	H
	Tectorigenin	OH	OCH_3_	OH	H	OH	H
	Irigenin	OH	OCH_3_	OH	OH	OCH_3_	OCH_3_
	Iristectorigenin A	OH	OCH_3_	OH	OH	OCH_3_	H
	Iristectorigenin B	OH	OCH_3_	OH	OCH_3_	OH	H
	Iridin	OH	OCH_3_	OGlc	OH	OCH_3_	OCH_3_
	Tectoridin	OH	OCH_3_	OGlc	H	OH	H
	Iristectorin A	OH	OCH_3_	OGlc	H	OCH_3_	OH
	Iristectorin B	OH	OCH_3_	OGlc	OCH_3_	OH	H

### Analysis of DEGs and Organ-Specific Genes (OSGs)

**FIGURE 4:**
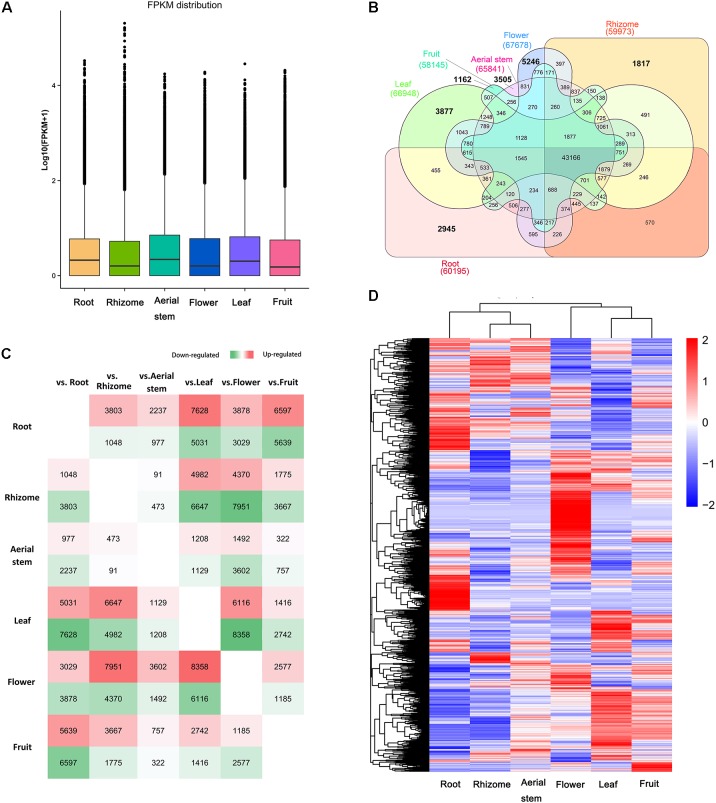
**(A)** Box plot showing fragments per kilobase of exon model per million mapped reads (FPKM) distribution of unigenes in each organ. **(B)** Venn diagram of expressed unigenes in each organ. The numbers in bold represent the number of organ-specific genes in the organs. **(C)** Number of up-regulated and down-regulated differentially expressed genes (DEGs) of each comparison between two organs. Green indicates down-regulated DEGs and red indicates up-regulated DEGs. The greener/redder the box is, the more DEGs in it. **(D)** Heat map showing gene expression patterns of each organ. The FPKM of each unigene among the six organs was normalized to draw the heatmap. Red indicates high relative gene expression level and blue indicates low relative gene expression level.

To investigate gene expression levels among the six organs, we mapped the high-quality reads from each sample onto the *B. chinensis* transcriptomes using RSEM. Clean reads from each organ were mapped back onto the assembled transcripts in *B. chinensis* using bowtie 2. To assess gene expression abundance, the expression levels of unigenes were measured by FPKM values. The distributions of expression levels in each organ are shown in **Figure 4A**. There were a total of 90,158 unigenes expressed above the threshold level (FPKM > 0.3) in the six organs. The flower had the highest number of expressed genes (67,678), followed by leaf (66,948), aerial stem (65,841), root (60,195), rhizome (59,973), and fruit (58,145) (**Table 1**). The highest number (14,474) of DEGs was in the flower followed by the leaf with 6116 highly expressed DEGs. Few DEGs were detected in the rhizome (564) and the aerial stem had 91 up-regulated unigenes. Among all DEGs, we identified 18,552 OSGs that were expressed in one organ only and 43,166 common unigenes that were expressed in all six organs. The overview of expressed unigenes, OSGs, and DEGs of each comparison is shown in **Figures 4B,C**. Global expression patterns deriving from the six organs using hierarchical cluster analysis of FPKM are shown in **Figure 4D**, indicated substantial transcriptional differences between the organs. Considering that the rhizome is the main source of crude drugs for medicinal use and contained the greatest abundance of isoflavonoids, comparative analysis between the rhizome and the other five organs was further investigated. The majority of DEGs were expressed at a lower level in the rhizome. Only 13 common DEGs were more expressed in the rhizome and 37 were less expressed (**Supplementary Figure 2**).

The GO enrichment analysis of DEGs was performed using 15 pairwise comparisons between organs to explore the major functional categories represented among them and to better understand the molecular mechanisms of different organs. “Metabolic process” was enriched with the most number of DEGs in all comparisons, followed by “catalytic activity,” “cellular process,” “binding,” “organic substance metabolic process,” and “primary metabolic process.” “Heme binding,” “tetrapyrrole binding,” “catalytic activity,” “peroxidase activity,” and “obsolete peroxidase reaction” were the top five significant terms in root compared to other organs. In the rhizome, “nutrient reservoir activity” was one of the most significant terms compared with other organs except stems. Notably, terms concerning regulation of many physiological processes were distinctively featured in rhizome. More details are shown in **Supplementary Table 5**. We also performed KEGG analyses on DEGs among the six organs (**Supplementary Figure 3**). The results showed that higher expressed DEGs in the root were enriched in “phenylpropanoid biosynthesis”; “plant–pathogen interaction”; “phenylalanine metabolism”; “stilbenoid, diarylheptanoid, and gingerol biosynthesis”; “plant hormone signal transduction”; and “starch and sucrose metabolism” compared with other organs. In the rhizome, the higher expressed genes were prominently enriched in “plant hormone signal transduction,” “flavonoid biosynthesis,” and “carbon fixation in photosynthetic organisms” compared with leaf, flower, and fruit; and “starch and sucrose metabolism” and “protein processing in endoplasmic reticulum” were most enriched compared with the root. In aerial stem, higher expressed unigenes were most prominently aligned to “photosynthesis” and “photosynthesis-antenna proteins” compared with root, leaf, flower, and rhizome; and “phenylpropanoid biosynthesis,” “flavonoid biosynthesis,” and “plant hormone signal transduction” were enriched compared with fruit, flower, and leaf. In the leaf, “photosynthesis,” “photosynthesis-antenna proteins,” “porphyrin and chlorophyll metabolism,” and “carbon fixation in photosynthetic organisms” were most representative pathways compared with root, rhizome, stem, and flower. In the flower, “starch and sucrose metabolism” and “pentose and glucuronate interconversions” were most representative compared with the other five organs. In fruit, “photosynthesis,” “photosynthesis-antenna proteins,” “porphyrin and chlorophyll metabolism,” and “carbon fixation in photosynthetic organisms” were most prominent compared with root, rhizome, and flower.

To further investigate the organ-specific biological processes, the annotations of the OSGs were manually screened (**Table 2**). Of root-specific genes, 54.6% were annotated, but up to 77.7% OSGs in aerial stem were not annotated in the seven databases, showing that these OSGs were *Belamcanda*-unique and remained unknown to date. The GO and KEGG annotations of OSGs in six organs displayed quite different classification patterns (**Supplementary Table 6**). “Integral component of membrane,” “membrane,” “DNA binding,” “protein binding,” “regulation of transcription,” and “oxidation–reduction process” were common representative terms in the six organs. “Regulation of transcription,” “nucleic acid binding,” “DNA integration,” and “nutrient reservoir activity” were distinctive terms in the rhizome; and in fruit “proteolysis” and “cell wall” were featured terms. For KEGG annotation, most OSGs were aligned to “metabolic pathways” and “biosynthesis of secondary metabolites” in all organs. “MAPK signaling pathway-plant,” which plays pivotal roles in the processes of response to various stimuli, was a featured pathway in the root. Three OSGs in leaf were clustered into “ABC transporters,” which serves in transport of a wide variety of substrates. “Cutin, suberin, and wax biosynthesis,” and “plant hormone signal transduction” were specifically representative in the flower.

### Gene Expression Analysis of (Iso)Flavonoid Pathway and Putative TFs in *B. chinensis*

**FIGURE 5:**
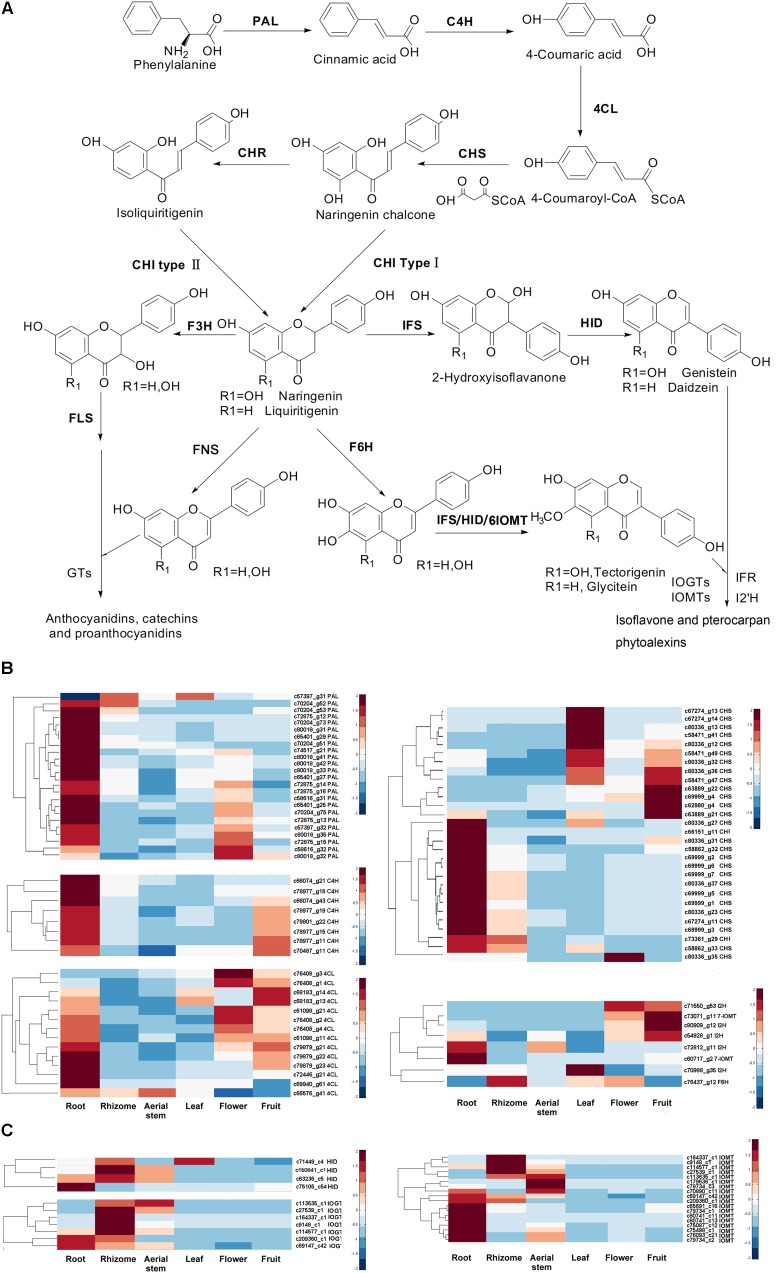
**(A)** Isoflavonoid pathway in legumes. PAL, phenylalanine ammonia lyase; C4H, cinnamate 4-hydroxylase; 4CL, *p*-coumaroyl coenzyme A ligase; CHS, chalcone synthase; CHI, chalcone isomerase, CHR, chalcone reductase; IFS, isoflavone synthase; HID, 2-hydroxyisoflavanone dehydratase; I2′H, isoflavone 2′-hydroxylase; FLS, flavonol synthase, FNS: flavone synthase, F3H, flavonone-3-hydroxylase; F6H, flavonoid 6-hydroxylase; IFR, isoflavone reductase; IOMT, isoflavone *O-*methyltransferases; IOGT, isoflavone *O-*glucosyltransferase; GTs, glucosyltransferases. **(B)** Expression level of genes which were annotated and involved in (iso)flavonoid pathway by KEGG database. The FPKM of each unigene among the six organs was normalized to draw the heatmap. Red indicates high relative gene expression level and blue indicates low relative gene expression level. **(C)** Expression level of HIDs, IOMTs, and IOGTs annotated in Swiss-Prot database. Red indicates high relative gene expression level and blue indicates low relative gene expression level.

In legumes, isoflavonoids are synthesized from *L*-phenylalanine via the isoflavonoid branch of phenylpropanoid metabolism (**Figure 5A**). However, we do not know if the isoflavonoid pathway in *B. chinensis* is similar to those of legumes. In this study, we investigated the expression patterns of genes involved in the isoflavonoid pathway among each organ of *B. chinensis* using the results of gene annotation, expression quantification, and differential analysis. We identified many unigenes encoding enzymes contributing to (iso)flavonoid biosynthesis from the *B. chinensis* transcriptome except those encoding IFS, HID, and chalcone reductase. Most genes had several isoforms. The KEGG annotation assigned 762, 192, and 20 unigenes to “phenylpropanoid biosynthesis,” “flavonoid biosynthesis,” and “isoflavonoid biosynthesis,” respectively. The greatest number of genes had their highest transcriptional level in the root (**Figure 5B**). PALs, C4H, CHSs, and CHIs had their lowest expression levels in the aerial stem. Most PALs had their highest expression in the root, followed by flower; and only one PAL isoform (c57397_g31) was abundant in the rhizome and leaf rather than root. The C4Hs were highly expressed in the root and fruit. The 4CL isoforms had their highest expression in the root, followed by flower and fruit. The CHSs had different expression patterns, with 16 unigenes expressed at their highest level in the root, followed by leaf and fruit. Isoform c80336_g35 had a high level in the flower, which differed from other CHS isoforms. In the isoflavonoid pathway, two putative isoflavone *O-*methyltransferases (IOMTs) were expressed in different modes: c60717_g2 was rich in the root while c73071_g11 was mainly distributed in the fruit. The putative flavonoid 6-hydroxylase (F6H) showed a special pattern of high expression level in the rhizome and flower. Seven CHS isoforms (c67274_g13, c67274_g14, c80336_g13, c58471_g41, c80336_g12, c58471_g49, and c80336_g32) had their highest expression levels in the leaf. Two CHI isoforms were higher expressed in the root and rhizome. In conclusion, the phenylpropanoid biosynthesis pathway has a higher relative gene expression level in root, fruit, and flower and the flavonoid biosynthesis pathway expressed higher in root, rhizome, flower, and fruit.

Isoflavonoid biosynthesis is a branch of flavonoid pathway. The flux of isoflavonoids is affected by genes controlling/regulating flavonoid pathway, the upstream pathway of flavonoid pathway (for example, phenylpropanoid pathway) and the competitive pathway (for example, flavonol pathway). There are many structural genes and regulatory genes contributing to the accumulation of isoflavonoids. To better understand the main influential factor of accumulation of isoflavonoids in different organs, we searched the Swiss-Prot database and found four unigenes (c63236_c5, c75105_c54, c71449_c4, and c150841_c1), which showed similarity to HID genes. HID, the second enzyme in the isoflavonoid biosynthesis pathway, is a critical determinant of isoflavone productivity in lotus hairy roots (Shimamura et al., 2007). The four putative HIDs showed higher expression gene level in rhizome, root, aerial stem, and leaf, which was corresponding to the distribution of isoflavonoids in each organ. Although we didn’t obtain IFS gene of *B. chinensis*, the expression level of HID may reflect the different accumulation of isoflavonoids in the six organs. In additional, we found putative IOGTs and IOMTs by BLAST against the Swiss-Prot database and screened several of them by comparing their expression level in different organs. The selected putative IOGTs have higher relative expression level in the rhizome and aerial stem (**Figure 5C**), which may contribute to the higher contents of glycosides in the rhizome and aerial stem rather than root. The selected putative IOMTs were mainly expressed in the root and rhizome and were expressed at higher levels in the root. It was consistent with the result that more irilone and dichotomitin (with more methoxyl groups) were detected in the root.

**FIGURE 6:**
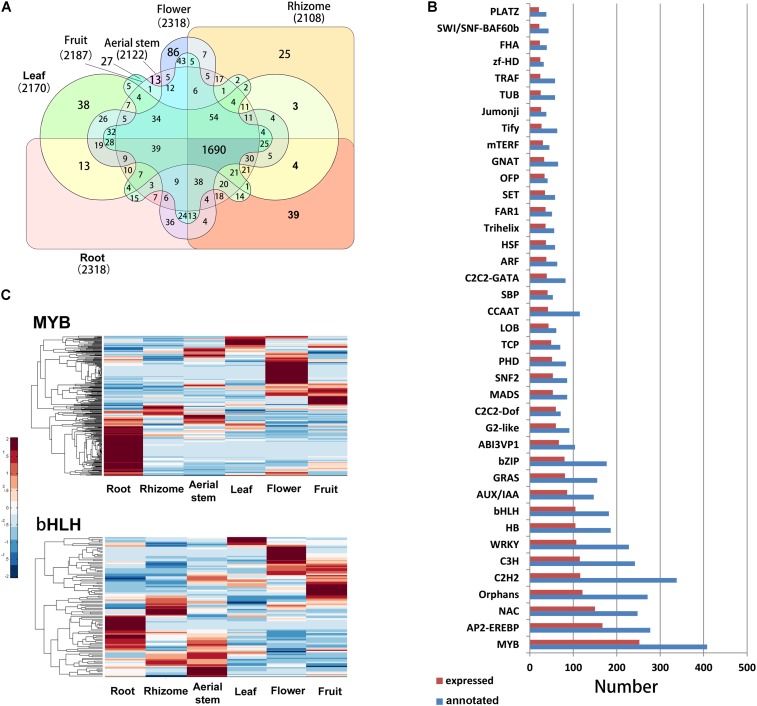
**(A)** Number of annotated and expressed TFs in each TF family. The families with more than 20 members were shown. **(B)** Venn diagram of expressed TFs in each organ. **(C)** Expression levels of putative MYB and bHLH genes in each organ. The FPKM of each unigene among the six organs was normalized to draw the heatmap. Red indicates high relative gene expression level and blue indicates low relative gene expression level.

The TFs play important roles in regulation of development, response to environment, and biosynthesis of secondary metabolites in plants. In our study, a total of 4995 unigenes were identified as TFs belonging to 82 TF families. Of the annotated TFs, 2817 were expressed (FPKM > 0.3) in at least one organ and 1690 were commonly expressed in all six organs. The root and flower possessed the most TFs and the rhizome had the least (**Figure 6A**). Among the 82 TF families, the MYB, AP2/EREBP, NAC, Orphans, C2H2, C3H, and WRKY families had the most members (**Figure 6B**). In *B. chinensis* transcriptomes, 408 MYB, 182 bHLH, 277 AP2/ERF, and 228 WRKY unigenes were identified. Many genes of MYB and bHLH subfamilies play vital roles in regulating flavonoid biosynthesis (Hichri et al., 2011; Xu et al., 2015). In *B. chinensis*, most MYB unigenes had higher expression levels in the root, followed by flower (**Figure 6C**). The bHLH unigenes showed high expression levels in the root, aerial stem, flower, and fruit.

### Identification and Homology Analysis of CYP450s in *B. chinensis*

**FIGURE 7:**
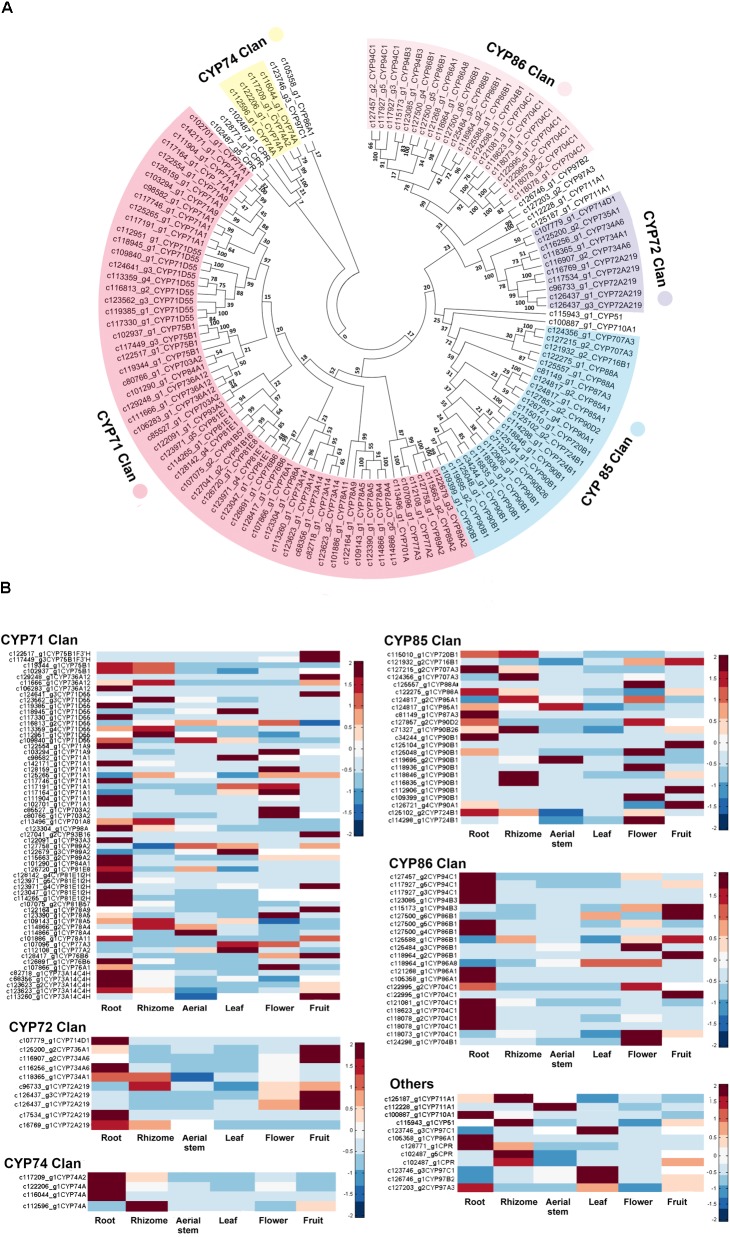
**(A)** Evolutionary relationships of identified cytochrome P450 proteins in *B. chinensis*. The evolutionary history was inferred using the Neighbor-Joining method. The optimal tree with the sum of branch length = 35.80740187 is shown. **(B)** Expression levels of CYP450s in each organ. The FPKM of each unigene among the six organs was normalized to draw the heatmap. Red indicates high relative gene expression level and blue indicates low relative gene expression level.

A total of 129 CYP450 proteins, ranging in size of 121–703 amino acids, were identified and clustered to 10 clans, of which there were four multi-family clans (CYP71s, CYP72s, CYP85s, and CYP86s) and six isolated clans: NADPH-dependent cytochrome P450 reductase (CPR), CYP51, CYP74, CYP97, CYP710, and CYP711. Multi-family clans had significantly more members in *B. chinensis* and the highest numbers of CYP450s were aligned to CYP71 clan (60 unigenes), followed by CYP85 (23), CYP86 (21), and CYP72 (10) clans. The CYP71 clan comprised 15 subfamilies: CYP71, CYP73, CYP75, CYP76, CYP77, CYP78, CYP81, CYP 84, CYP 89, CYP91, CYP 93, CYP98, CYP701, CYP703, and CYP736. The CYP85 clan had eight subfamilies: CYP85, CYP87, CYP88, CYP90, CYP707, CYP716, CYP720, and CYP724. Subfamilies CYP86, CYP94, and CYP704 belonged to CYP86 clan; and CYP72, CYP714, CYP734, and CYP735 belonged to CYP72 clan. Most CYP450s belonged to the same clan gathered in the same branch except for c123746_g3 (CYP97) and c105358 _g1 (CYP86B) in the inferred phylogenetic tree (**Figure 7A**) and CYP97 clan was close to the CYP86 clan. The CYP74 clan and CPR were grouped far from other clans.

In plants, CYP450s often catalyze diverse biosynthetic reactions, most of which are monooxygenation reactions and hydroxylation reactions producing various metabolites, including alkaloids, terpenes, phenolics and their conjugates, flavonoids, coumarins, lignans, and glucosinolates. CYP450s also play important roles in the homeostasis of plant hormones and signaling molecules participating in the defensive and developmental processes. The number and diversity of CYP450 genes make a lot of sense for the biosynthesis of bioactive compounds and other physiological process in plants. In our work, a total of 129 CYP450s, from 35 families of 10 clans were identified. CPR is a membrane bound protein localized in the ER membrane. CYP707A encoding abscisic acid 8′-hydroxylase is a key enzyme in abscisic acid catabolism (Krochko et al., 1998). CYP701 family encoding ent-kaurene oxidase and CYP88A encoding ent-kaurenoic acid oxidase are required for gibberellin biosynthesis (Helliwell et al., 2001; Wang et al., 2012). CYP724, CYP90 and CYP85, and CYP90A/B families catalyze essential oxidative reactions in the biosynthesis of brassinosteroid hormones which regulate numerous plant processes, including cell elongation, vascular differentiation, and so on (Ohnishi et al., 2006; Sakamoto et al., 2012). CYP710, encoding Sterol C-22 Desaturase, is closely located near CYP51 (Sterol 14α-demethylase or obtusifoliol 14α-demethylaseas) and both of them contribute to sterol biosynthesis (Morikawa et al., 2006; Lepesheva and Waterman, 2007). CYP74A can encode allene oxide synthase and catalyze the precursor of jasmonic acid (Laudert et al., 1996) and CYP94B/C can catalyze two reactions of phytohormone jasmonoyl-isoleucine which regulates plant growth, development, and immune function (Koo et al., 2011; Heitz et al., 2012). CYP73A, CYP85E, CYP81, and CYP93 are reported to take part in flavonoid pathway. The CYP73A subfamily encoding C4H could catalyze the second step in the phenylpropanoid pathway. CYP75A encoding flavonoid 3′,5′-hydroxylase and flavonoid 3′-hydroxylase was involved in the biosynthesis of anthocyanin pigments (Tanaka, 2006). The CYP81E subfamily encoding isoflavone 2′- and 3′-hydroxylase and the CYP93A subfamily encoding 3,9-dihydroxypterocarpan 6a-hydroxylases (Schopfer and Ebel, 1998) are reported to participate in phytoalexin biosynthesis—a downstream branch of the isoflavonoid pathway. However, CYP93C unigene encoding IFS was not identified in *B. chinensis* transcriptomes.

The relative gene expression levels of each CYP450s were also analyzed and shown in **Figure 7B**. Unigenes from same subfamily may show same distribution patterns or not. It was related to their specific physiological functions in *B. chinensis*.

### Quantitative Real-Time PCR

There were 14 selected unigenes chosen to validate the differential expression data obtained by RNA-Seq. Each pair of primers had good specificity and amplification efficiency around 100%. The results showed good correlations for each unigene between the measured relative quantity by real-time PCR and FPKM calculated from RNA-Seq data (**Supplementary Figure 4**), confirming the reliability of RNA-Seq data.

### Cloning of Genes Annotated as Participating in (Iso)Flavonoid Biosynthesis

**FIGURE 8:**
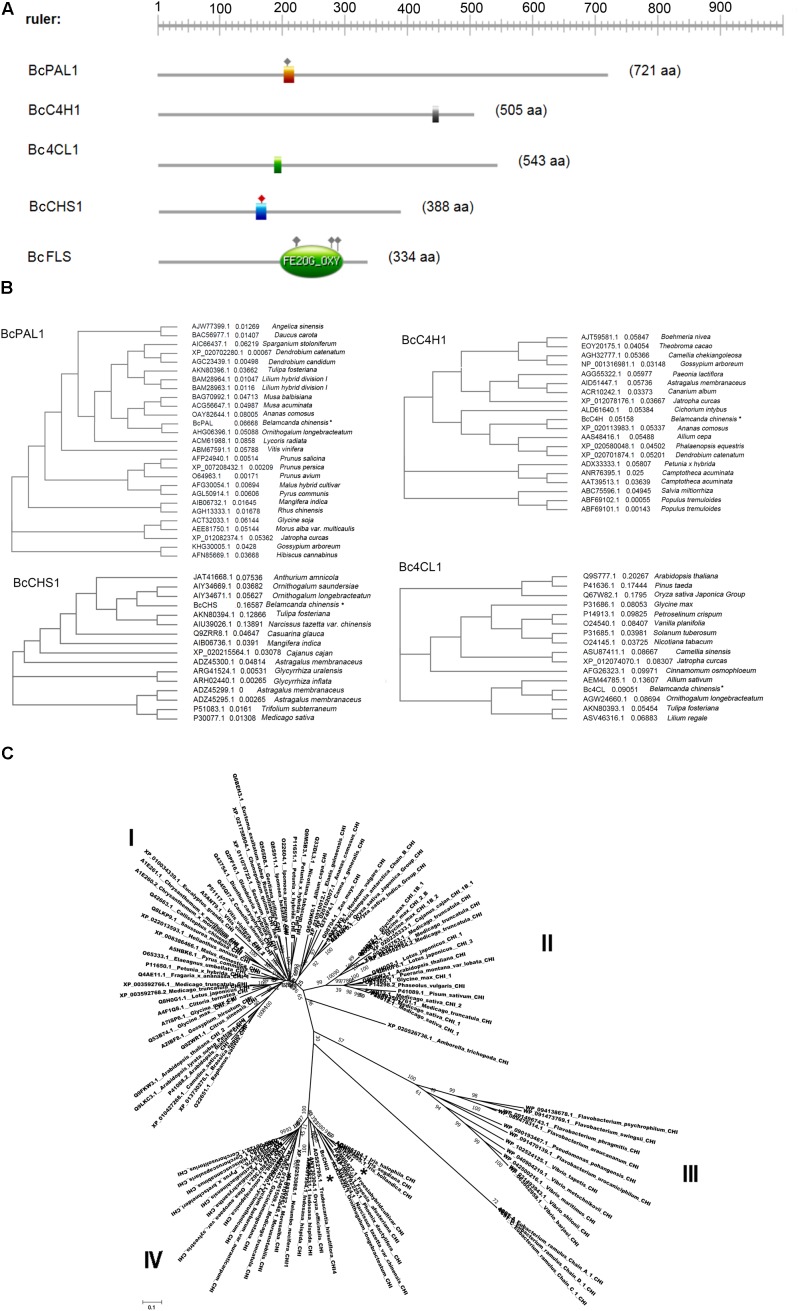
**(A)** The graphical representations of domains, their colors, and shapes are not intended to indicate homology or shared function. **(B)** Phylogenetic trees of cloned proteins with their homologs of other plants. PAL, phenylalanine ammonia lyase; C4H, cinnamate 4-hydroxylase; 4CL, *p*-coumaroyl coenzyme A ligase; CHS, chalcone synthase; CHI, chalcone isomerase. “^∗^” points the proteins of *B. chinensis*. **(C)** Evolutionary relationships of CHI proteins in plants and bacteria. The evolutionary history was inferred using the Neighbor-Joining method. The optimal tree with the sum of branch length = 19.64834842 is shown. The tree is drawn to scale, with branch lengths in the same units as those of the evolutionary distances used to infer the phylogenetic tree. The evolutionary distances were computed using the Poisson correction method and are in the units of the number of amino acid substitutions per site. The analysis involved 110 amino acid sequences. All ambiguous positions were removed for each sequence pair. Evolutionary analyses were conducted in MEGA7. “^∗^” points the proteins of *BcCHI1* and *BcCHI2*.

We successfully cloned six enzyme genes: *BcPAL1, BcC4H1, Bc4CL1, BcCHS1, BcCHI1*, and *BcCHI2* (Accession Nos.: MF979129, MF979130, MG256475, MF979131, MF979132, and MF979133). The cloned genes showed high similarity (99–100%) to assembled unigenes. The parameters (including molecular weight, PI, grand average of hydropathicity, aliphatic index, instability index, and transmembrane helices) of the encoded proteins are shown in **Table 3**. *BcC4H* is the only basic protein. The *Bc4CL2* is a hydrophobic protein with a high aliphatic index. *BcCHI1* is relatively stable among the six proteins. All the six proteins are outside the membrane. The domains predicted by ExPASy-PROSITE tool were shown in **Figure 8A**. Homology analysis was performed using NCBI BLAST tool (BLASTX 2.6.0+^10^). *BcPAL1, BcC4H1, Bc4CL1*, and *BcCHS1* showed high homology with representatives in the Swiss-Prot database, which provides high-quality annotated proteins. *BcCHI1* and *BcCHI2* had 66% similarity to CHI of *Arabidopsis thaliana* [Q8VZW3]. The amino acid sequences of *BcPAL1, BcC4H1, Bc4CL1*, and *BcCHS1* were separately aligned with their homologous proteins from other plants (**Figure 8B**). They showed high homology with representatives from monocots. *BcPAL1, Bc4CL1*, and *BcCHS1* had the highest homology with proteins from *Ornithogalum* (Liliaceae) and *BcC4H1* was most homologous to C4H of *Ananas* (Bromeliaceae). *BcCHI1* and *BcCHI2* had most similarity with CHIs of *Iris* and *Freesia* (Iridaceae) in Nr database. By constructing a phylogenetic tree with 108 protein sequences of CHI, 94 of which are from plants and 14 of which are from microorganisms, *BcCHI1* and *BcCHI2* were clustered into the type IV branch (**Figure 8C**), which doesn’t catalyze the formation of *S*-flavanone and was proved as an enhancer of flavonoid production (Ngaki et al., 2012; Morita et al., 2014; Jiang et al., 2015). This study provides a good foundation for further study on the (iso)flavonoid biosynthesis pathway in *B. chinensis*.

## Discussion

### DEGs May Play Crucial Roles in Organ Function and Morphogenesis in *B. chinensis*

Higher plants comprise several organs made up of various tissues and cell types. Gene expression patterns differed among the organs of *B. chinensis* in our study. To better reveal the genes associated with organ morphogenesis and function, comparative expression analysis of the transcriptomes among organs was performed. Both DEGs and OSGs were subjected to GO and KEGG annotation. The results promoted our understanding of the gene expression patterns among organs. Most DEGs occurred in basic functions such as metabolic process, catalytic activity, cellular process, single-organism process, binding, and organic substance metabolic process. In the root, DEGs were significantly enriched in pathways of “phenylpropanoid biosynthesis,” “phenylalanine metabolism,” “plant–pathogen interaction,” “flavonoid biosynthesis,” “starch and sucrose metabolism,” and “plant hormone signal transduction,” suggesting the defense and plant hormone signaling function of the root. Strikingly, the rhizome differed from root with higher enriched function of “flavonoid biosynthesis” and “plant hormone signal transduction,” suggesting that the rhizome contributed to flavonoid biosynthesis and regeneration. In rhizome, GO terms of “nutrient reservoir activity” and regulation and negative regulation of many physiological processes were most enriched, corresponding to the functions of storing nutrients and regulation. Compared to underground organs, aboveground organs had more representatives in “photosynthesis, photosynthesis-antenna proteins,” and “porphyrin and chlorophyll metabolism,” which are related to photosynthesis. Root-specific unigenes consistently exhibited apparent expression in an organ-dependent manner. A functional analysis using the GO classification system revealed that each group of OSGs was associated with various characteristic GO terms.

### Isoflavonoid Accumulation in *B. chinensis*

In our previous report, we had found that isoflavones and their glycosides accumulated in different tissues of *B. chinensis* rhizome (Chen et al., 2014). Quantitative analysis of isoflavonoids in different organs in the present study showed that the root, rhizome, and aerial stem contained different amounts of various isoflavonoids while leaf, flower, and fruit accumulated few. Distinctively, the rhizome had significantly higher levels of total isoflavonoids compared with other organs and accumulated more glycosides than their aglycones, which might signify the essential roles of isoflavonoids in plant development and physiology.

### Isoflavonoid Biosynthesis in *B. chinensis*

The phenylpropanoid and flavonoid pathways are general metabolic processes in higher plants and have been thoroughly illustrated in various plant species from different families. The isoflavonoid pathway has been demonstrated almost exclusively in legumes. In our work, a large number of genes involved in the (iso)flavonoid pathway, TFs (MYBs and bHLHs), and CYP450s were identified in *B. chinensis*. In this plant, the phenylpropanoid and flavonoid pathways were revealed with sufficient annotated unigenes. However, downstream of the isoflavonoid pathway was annotated with few predicted unigenes: 17 I2′H genes, two isoflavone-7-*O-*methyltransferase genes, one isoflavone 7-*O-*glucoside-6’-*O-*malonyltransferase gene, and one F6H gene. This may be due to the limited amount of reference sequences, which were also exclusive to leguminous plants. The four putative HIDs may contribute to the accumulation of isoflavonoids in different organs. The IOMTs and IOGTs may contribute to the distribution of specific isoflavone. Some members of the MYB family were confirmed to regulate or affect isoflavonoid biosynthesis in soybean (Yi et al., 2010; Chu et al., 2017; Zhao et al., 2017). The annotated 408 putative MYBs and 182 putative bHLHs in *B. chinensis* transcriptomes can be further screened to discover candidates with activity of regulating isoflavonoid biosynthesis. Among the CYP450 families identified in this work, subfamilies of CYP73A, CYP85E, CYP81, and CYP93 were reported to take part in the flavonoid pathway.

Isoflavone synthase belongs to the CYP93C subfamily and is the first enzyme that catalyzes the formation of isoflavone skeleton and plays a crucial role in biosynthesis of isoflavonoids. During 2000–2017, IFS were identified from 23 leguminous plants and 1 non-leguminous plant *Beta vulgaris* (Chenopodiaceae) and showed high degrees (>78%) of similarity at the protein level. None of IFS in other plant taxa has been reported so far. Lapcik et al. (2006) detected the presence of isoflavonoids in *A. thaliana* and examined genes in public transcriptomic datasets of *A. thaliana*; however, no homologous IFS genes were found. As a result, they concluded that another gene might be responsible for the biosynthesis of isoflavonoids in Brassicaceae.

Despite *B. chinensis* containing large amounts of various isoflavonoids and that 423,661 unigenes were obtained from the comprehensive transcriptome of *B. chinensis*, we confronted the same problem as Lapcik et al. (2006). No unigene was annotated as a homolog of known IFS genes in *B. chinensis* transcriptomes. Thus, we speculated that IFS in *B. chinensis* under general physiological conditions might be expressed at a level below the detection limit of the Illumina HiSeq 2500 platform. Another speculation was that the enzyme catalyzing the formation of the isoflavonoid skeleton in *B. chinensis* has a quite low homology with IFS in legumes. To solve these puzzles, we suggest that elicitors should be screened to stimulate the plant to give rise to isoflavonoid biosynthesis so that comparative analysis of the transcriptome can be applied to discover the IFS and other genes involved.

### Distinctive Hydroxylation, O-Methylation, and O-Glycosylation of Belamcanda-Isoflavones

Most *Belamcanda*-isoflavones are characterized by 6-methoxylation (**Table 4**). Glycitein, one of the major isoflavone aglycones in soybean, is structurally similar to tectorigenin except for the absence of a 5-hydroxyl moiety. In *Glycine max*, glycitein is known to be synthesized via a key enzyme F6H. The C6-hydroxylation of the A-ring of isoflavone is likely to occur at the flavanone, rather than the isoflavone level, because F6H (CYP71D9) catalyzes conversion of flavanones more efficiently than flavones; whereas isoflavones are hardly 6-hydroxylated and IFS can catalyze 6,7,4′-trihydroxyflavanone efficiently (Latunde-Dada et al., 2001). In addition, flavonol 6-hydroxylase, a 2-oxoglutarate-dependent dioxygenase, was identified from *Chrysosplenium americanum* and shown to catalyze the 6-hydroxylation of partially methylated flavonols. However, it had no activity on flavones (Anzellotti and Ibrahim, 2000, 2004). In sweet basil and peppermint (*Mentha × piperita*), two novel flavone-6-hydroxylases belonging to the CYP82D subfamily were found to catalyze 6-hydroxylation of flavones or flavanones with 5-hydroxyl and 7-methoxyl residues (Berim and Gang, 2013). Recently, Zhao et al. (2018) found one flavone 6-hydroxylase (CYP82D1.1) from *Scutellaria baicalensis* with broad substrate specificity for flavones. The aforementioned three types of 6-hydroxylases of flavonoids share low homology with each other.

In our study, c76437_g12 was annotated as F6H in KEGG and Swiss-Prot databases with 71% similarity to F6H1 of *G. max* (Accession No.: O81971.1). The unigene c76437_g12 had the same expression levels in the rhizome and flower but lowest expression in the root and fruit of *B. chinensis*. The complete coding sequence and functional characterization should be further studied.

The methylation of isoflavonoids is catalyzed by IOMTs, which transfers a methyl group from *S*-adenosyl-*L*-methionine to a hydroxyl moiety of isoflavones. Methylation can alter the bioactivity and modulate solubility and intracellular localization of isoflavonoids (Ibrahim et al., 1987). The most common sites of methylation of isoflavonoids in legumes are the C4′- and 7-positions. To date, C3′-, 4′-, 5-, or 7-*O-*methylation activity of IOMTs has been characterized in several legumes (Khouri et al., 1988; He et al., 1998; He and Dixon, 2000; Liu and Dixon, 2001; Akashi et al., 2003; Deavours et al., 2006; Li et al., 2016a,b; Mochida et al., 2017). No isoflavone-6-*O-*methyltransferase has yet been found so far. Isoflavone glucosyltransferases contribute to the biosynthesis of glycosides of isoflavones. The C7- and 4′-position regiospecific isoflavone glucosyltransferases have been most investigated in *G. max* (Noguchi et al., 2007; Livingstone et al., 2011; Funaki et al., 2015), followed by *Medicago truncatula* (Li et al., 2007; Modolo et al., 2007), *Pueraria lobata* (Li et al., 2014; Wang et al., 2016), *Glycyrrhiza echinata* (Nagashima et al., 2004), and *Cicera rietinum* (Koster and Barz, 1981). In the present work, we screened 56 putative (iso)flavonoid *O*-methyltransferases and 16 putative (iso)flavonoid *O*-glucosyltransferases according to Pfam, KO, and Swiss-Prot annotation (**Supplementary Table 7**). In our following work, experimental characterization will be investigated to explore their specific functions.

## Conclusion

In this study, the high-quality transcriptome sequencing data of six organs of *B. chinensis* were obtained and the results of *de novo* assembly, functional annotation, and expression profile were presented. DEGs and OSGs were screened and analyzed to profile expression patterns of each organ, which will provide a better understanding of the functions of the organs. Furthermore, the TFs, CYP450s, and a host of structural genes involved in various metabolic pathways of the plant were identified, and we cloned six key enzyme genes associated with (iso)flavonoid biosynthesis in *B. chinensis*. To our knowledge, this is the first report of *de novo* transcriptome analysis of *B. chinensis* and it will provide a sound platform for characterization of genes associated with biosynthesis, metabolism, and regulation of secondary metabolites and metabolic engineering.

## Data Availability

Transcriptome data and assembled unigenes in this study have been deposited in the National Center for Biotechnology Information (NCBI) under the accession number PRJNA430284. The assembled sequences together with annotations are also freely available in figshare (10.6084/m9.figshare.5809827.v1).

## Author Contributions

MQ, YZ, and MT conceived the project and designed the experiments. MT, XZ, and GX performed the experiments. MT, XZ, and YZ analyzed the data and plotted the graphs. MT and MQ were responsible for manuscript drafting. All the authors carefully revised and approved this manuscript.

## Conflict of Interest Statement

The authors declare that the research was conducted in the absence of any commercial or financial relationships that could be construed as a potential conflict of interest.

## Footnotes

1 http://www.ncbi.nlm.nih.gov/COG

2 http://www.genome.jp/kegg/

3 http://www.genome.jp/kegg/kaas/

4 http://bioinfo.bti.cornell.edu/tool/itak

5 http://www.interactivenn.net/

6 http://drnelson.uthsc.edu/CytochromeP450.html

7 http://prosite.expasy.org/

8 http://web.expasy.org/compute_pi/

9 http://www.cbs.dtu.dk/services/TMHMM/

10 https://blast.ncbi.nlm.nih.gov/Blast.cgi
