# Supplementary material for: Global Transcriptome Analyses Reveal Differentially Expressed Genes of Six Organs and Putative Genes Involved in (Iso)flavonoid Biosynthesis in Belamcanda chinensis
Source: Front Plant Sci. 2018 Aug 14;9:1160. doi: 10.3389/fpls.2018.01160 (PMC6102373; doi:10.3389/fpls.2018.01160)
Supplement: Supplementary file 1 [file Table_1.DOCX]

**Supplementary Table 1. Primers used in this experiment.**

**A. Primers for cloning CDSs of genes involved in flavonoid biosynthesis**

|  | Primer 5'-3' |
| --- | --- |
| *BcPAL1* F | ATGGAGAACGGCAATGGCTAC |
| *BcPAL1* R | GGACTATTTCTCCTAACATATGGG |
| *BcC4H1* F | ATGGACGTGCGGCTTGTCG |
| *BcC4H1* R | TCAAAACACTCTAGGTTTGGCCACT |
| *Bc4CL1* F | ATGGGCTCCATCCCTTCCGAG |
| *Bc4CL1* R | TCAAAGGTGTTGATCGTTGGC |
| *BcCHI1* F | ATGGATTCTGAGATGGTGATGG |
| *BcCHI1* R | GAAAATTCTTATGCTGCTAGTTT |
| *BcCHI2* F | ATGGGTTCTGAAATGGTGATGGT |
| *BcCHI2* R | TTTTATGCTGCTAAATTCTCAGCGAG |
| *BcCHS1* F | ATGGGATCATCGAGTGAGGT |
| *BcCHS1* R | AGTTATGGACACCTAAGGAGAACT |

**B. Primers for validation of gene expression by real-time PCR**

| unigene ID | Primer 5'-3' | Length of product  /bp | Annotation |
| --- | --- | --- | --- |
| c61429_g32 F | TGCCATGTATGTCGCTATCCAG | 122 | Actin |
| c61429_g32 R | GTGCATATCCTTCATAAATTGGAAC |  |  |
| c73402_g51 F | GCTGATCGACTCCGTGCTC | 272 |  |
| c73402_g51 R | GGACCATACACTCATCAGCATTC |  |  |
| c61398_g25 F | ACATCGACAAATCTGCCTACGAG | 183 |  |
| c61398_g25 R | ACTCGCGGTAGCTCACCTTG |  |  |
| c76437_g12 F | CCTCTGGCGAGGAGACGACGAT | 319 | putative F6H |
| c76437_g12 R | CCCCCAGGGCCATGGAAGCT |  |  |
| c69867_g26 F | GCTGCGTCTCCGTCCAC | 222 | CHI isoform |
| c69867_g26 R | ACCGTTTGCTATGTGAACTCA |  |  |
| c69867_g24 F | TCCGTCCACAGTCAAGAGC | 162 | CHI isoform |
| c69867_g24 R | CGTGCAGTCTCCAACAGT |  |  |
| c80366_g51 F | TCAACTTCTACCCCAAGTGTCC | 451 | F3H |
| c80366_g51 R | CATTCTTGAACCTACCGTTGCT |  |  |
| c63889_g22 F | CCGCTGTTTCAACTGATATACGC | 202 | CHS |
| c63889_g22 R | GCCACCCAGAAAATCGAGT |  |  |
| c72812_g11 F | TGTACCCTCCGACTACGCATT | 200 | I’2H |
| c72812_g11 R | ACTCAACAACCCGGACAAGC |  |  |
| c57079_g12 F | GGCGATCTCTTTCTCTATCCC | 178 |  |
| c57079_g12 R | GCTGGATGCTTCTACCACT |  |  |
| c77609_g52 F | CGGACCAAAGCAAGATATCGAA | 176 |  |
| c77609_g52 R | ACACGCTTGTCAATGCC |  |  |
| c66527_g2 F | CCGGGGACACGACCCTTCCCGATT | 178 | CHR-like |
| c66527_g2 R | CCACCTGATTCACCGATGGAGGTATCC |  |  |
| c66074_g21 F | AGGCGAAGCTCGACACCT | 131 | C4H |
| c66074_g21 R | CGTCGTCACAAAGCTTCGTTC |  |  |
| c57043_g8 F | GCCCTCTTCGCGTACATCA | 194 |  |
| c57043_g8 R | CGCACCATCCAGTTCGT |  |  |
| c73361_g29 F | TGGCAATAAATTGACCGAAGTGAA | 149 | CHI isoform |
| c73361_g29 R | GTCCGAACAGACCGTCAATG |  |  |
