# Supplementary material for: Global Transcriptome Analyses Reveal Differentially Expressed Genes of Six Organs and Putative Genes Involved in (Iso)flavonoid Biosynthesis in Belamcanda chinensis
Source: Front Plant Sci. 2018 Aug 14;9:1160. doi: 10.3389/fpls.2018.01160 (PMC6102373; doi:10.3389/fpls.2018.01160)
Supplement: Supplementary file 3 [file Table_3.DOCX]

**Supplementary table 3 Overview of the quality of reads**

| **Sample** | **Raw Reads** | **Clean Reads** | **Clean Bases**  **(Gbp)** | **Error**  **(%)** | **Q20**  **(%)** | | **Q30**  **(%)** | | **GC Content**  **(%)** | |
| --- | --- | --- | --- | --- | --- | --- | --- | --- | --- | --- |
| Root1 | 52,881,136 | 51,201,380 | 7.68 | 0.02 | | 96.62 | | 91.79 | | 51.37 |
| Root2 | 55,400,466 | 51,939,630 | 7.79 | 0.02 | | 95.58 | | 89.50 | | 51.41 |
| Root3 | 51,033,822 | 50,690,034 | 6.34 | 0.02 | | 96.83 | | 91.97 | | 51.43 |
| Rhizome1 | 50,367,510 | 46,582,410 | 6.99 | 0.02 | | 95.89 | | 90.07 | | 51.48 |
| Rhizome2 | 41,761,274 | 39,417,530 | 5.91 | 0.02 | | 95.70 | | 89.70 | | 51.04 |
| Rhizome3 | 57,718,330 | 57,288,538 | 7.16 | 0.02 | | 96.81 | | 92.00 | | 49.93 |
| Aerial  stem1 | 59,108,850 | 55,526,578 | 8.33 | 0.02 | | 95.78 | | 89.90 | | 49.82 |
| Aerial  stem2 | 51,583,350 | 48,589,494 | 7.29 | 0.02 | | 95.55 | | 89.48 | | 50.85 |
| Aerial  stem3 | 51,676,516 | 51,234,912 | 6.40 | 0.02 | | 96.83 | | 92.12 | | 49.87 |
| Leaf1 | 48,673,516 | 47,068,958 | 7.06 | 0.02 | | 96.63 | | 91.80 | | 50.87 |
| Leaf2 | 48,460,880 | 46,798,366 | 7.02 | 0.02 | | 96.73 | | 92.00 | | 50.31 |
| Leaf3 | 52,051,610 | 51,666,890 | 6.46 | 0.02 | | 96.81 | | 91.99 | | 49.47 |
| Flower1 | 48,370,672 | 46,681,924 | 7.00 | 0.02 | | 96.71 | | 91.95 | | 49.90 |
| Flower2 | 59,111,860 | 56,571,238 | 8.49 | 0.02 | | 96.67 | | 91.85 | | 50.15 |
| Flower3 | 49,096,822 | 48,746,162 | 6.09 | 0.02 | | 96.77 | | 91.85 | | 49.54 |
| Fruit1 | 52,812,644 | 50,477,588 | 7.57 | 0.02 | | 96.86 | | 92.25 | | 50.96 |
| Fruit2 | 45,771,202 | 44,173,378 | 6.63 | 0.02 | | 96.63 | | 91.78 | | 53.03 |
| Fruit3 | 51,881,330 | 51,474,408 | 6.43 | 0.02 | | 96.45 | | 91.44 | | 50.00 |
| Total | 927,761,790 | 896,129,418 | 126.64 |  | |  | |  | |  |
