# Supplementary figures and images for: Global Transcriptome Analyses Reveal Differentially Expressed Genes of Six Organs and Putative Genes Involved in (Iso)flavonoid Biosynthesis in Belamcanda chinensis
Source: Front Plant Sci. 2018 Aug 14;9:1160. doi: 10.3389/fpls.2018.01160 (PMC6102373; doi:10.3389/fpls.2018.01160)

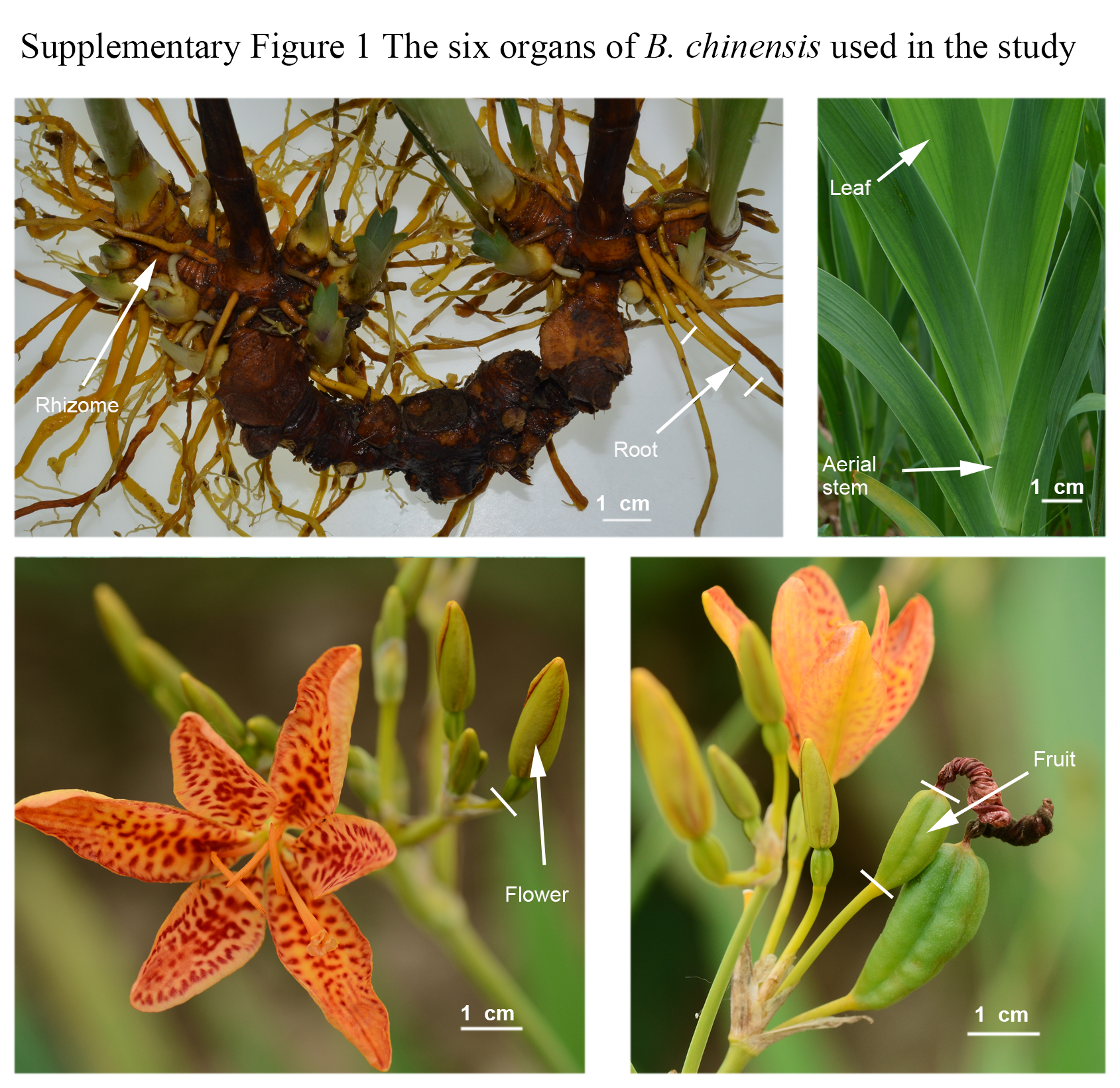

Supplement: Supplementary file 8 [file Image_1.tif]

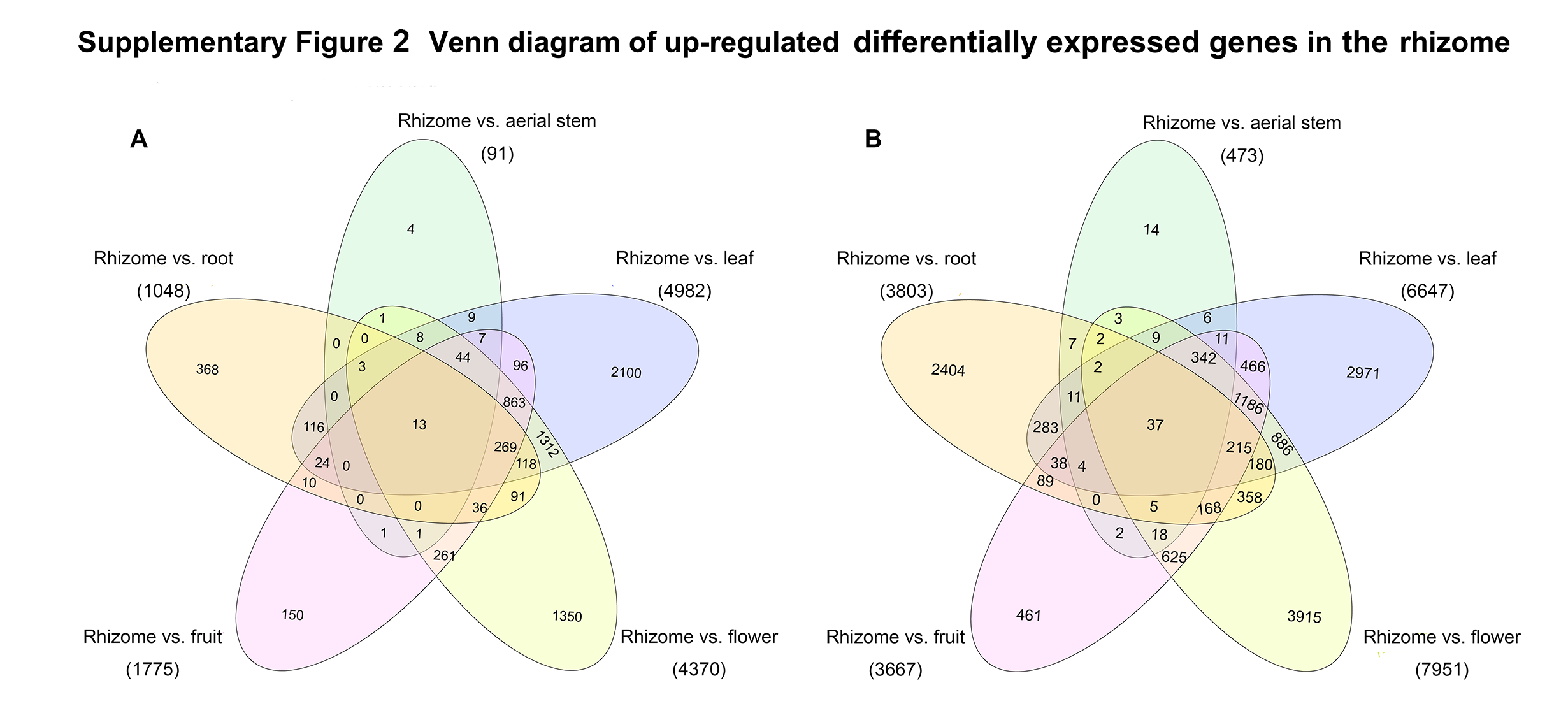

Supplement: Supplementary file 9 [file Image_2.TIF]

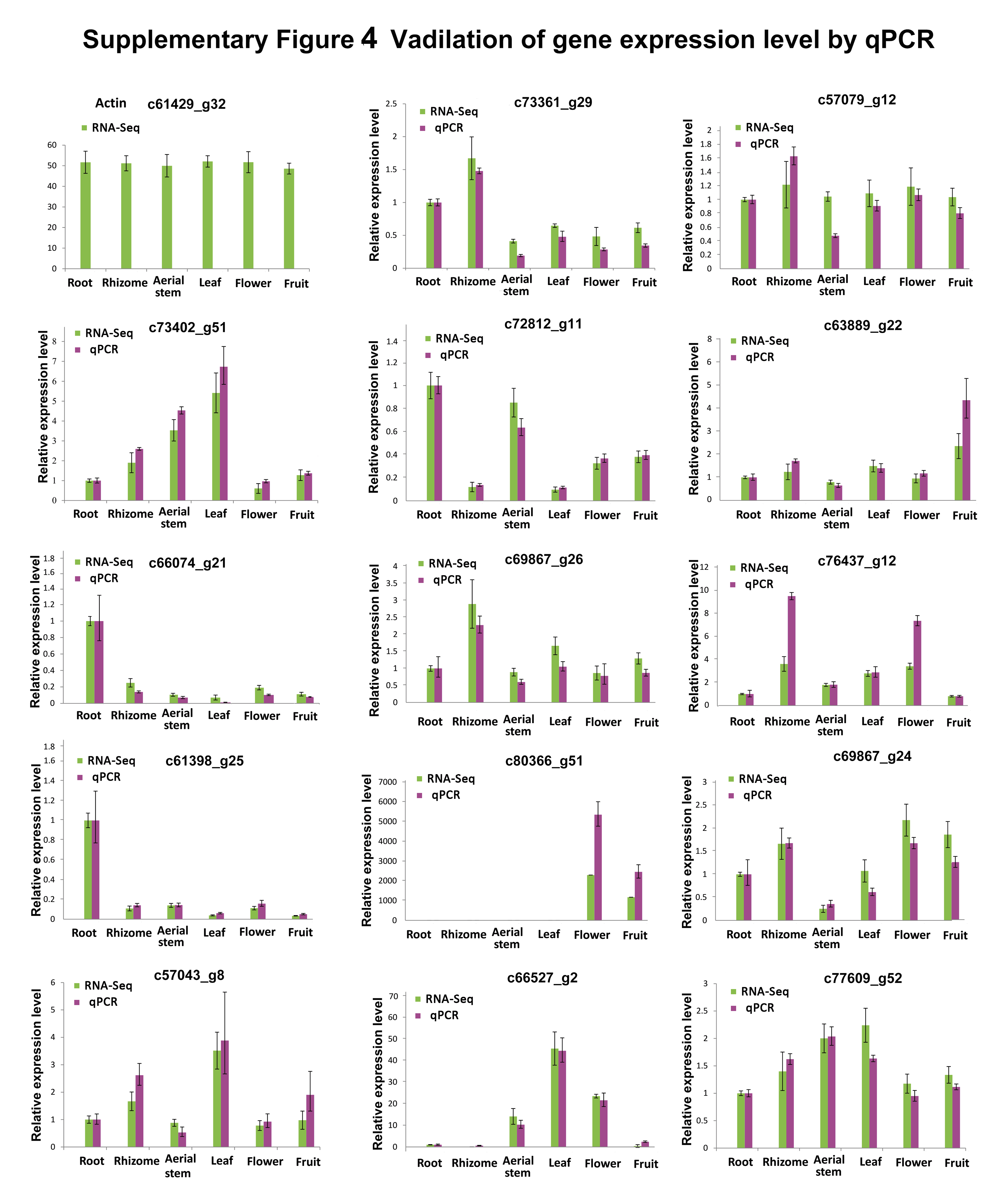

Supplement: Supplementary file 11 [file Image_4.TIF]
