# Supplementary material for: Global Transcriptome Analyses Reveal Differentially Expressed Genes of Six Organs and Putative Genes Involved in (Iso)flavonoid Biosynthesis in Belamcanda chinensis
Source: Front Plant Sci. 2018 Aug 14;9:1160. doi: 10.3389/fpls.2018.01160 (PMC6102373; doi:10.3389/fpls.2018.01160)

Supplementary Figure 3

A. Top 20 KEGG enrichment pathways of higher-expressed DEGs in the root.

Root vs. rhizome

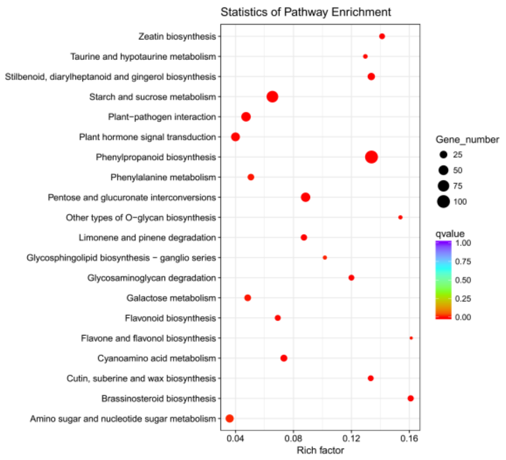

Root vs. fruit

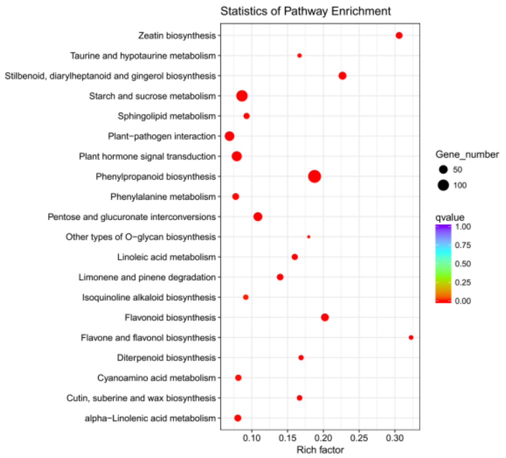

Root vs. stem

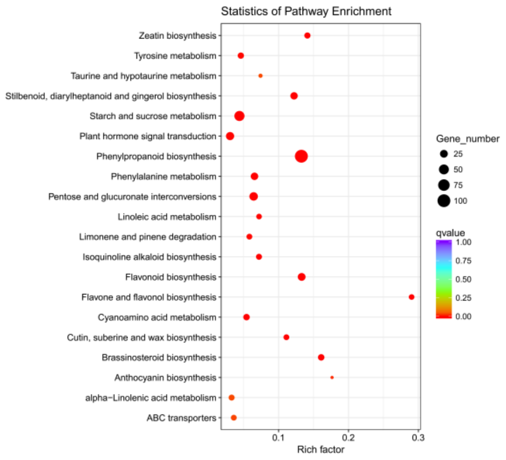

Root vs. leaf

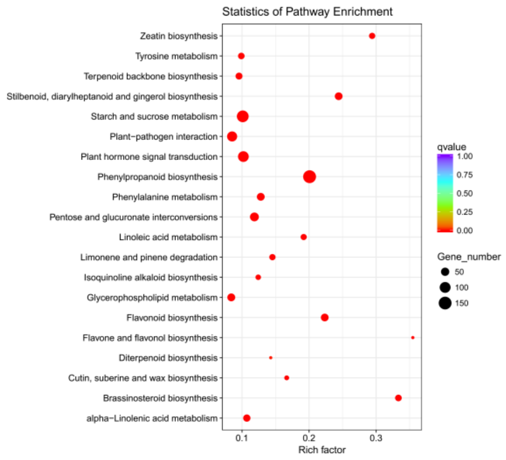

Root vs. fruit

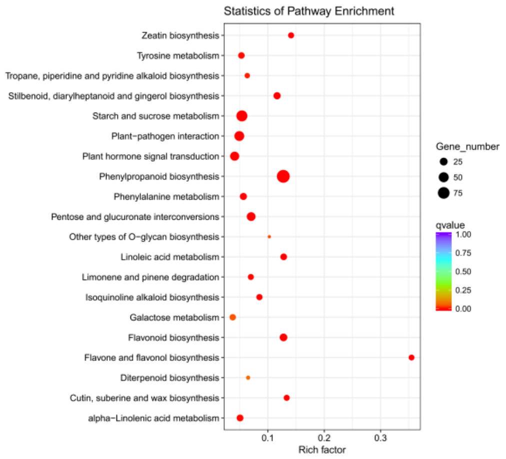

B. Top 20 KEGG enrichment pathways of higher-expressed DEGs in the rhizome.

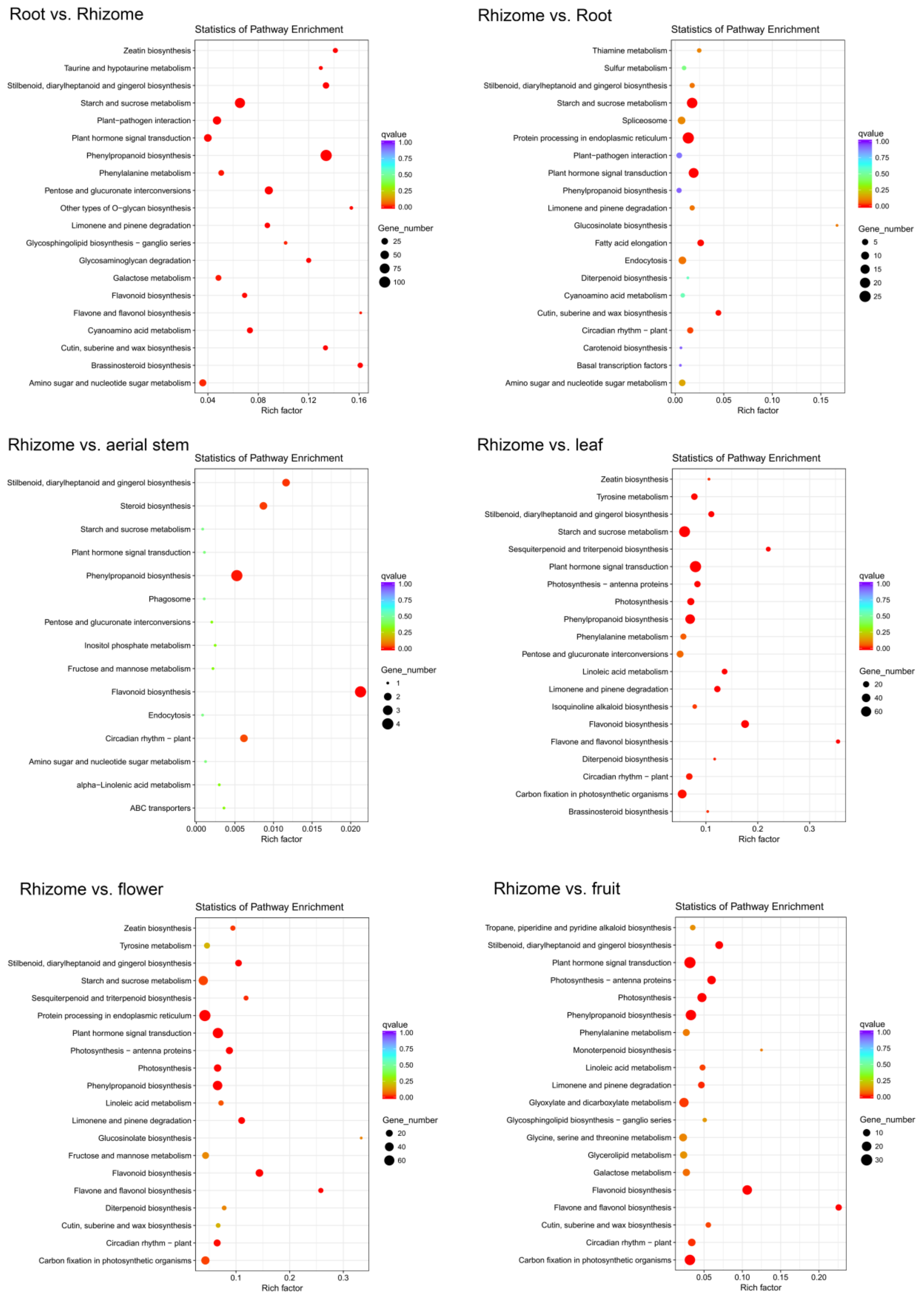

C. Top 20 KEGG enrichment pathways of higher-expressed DEGs in the aerial stem.

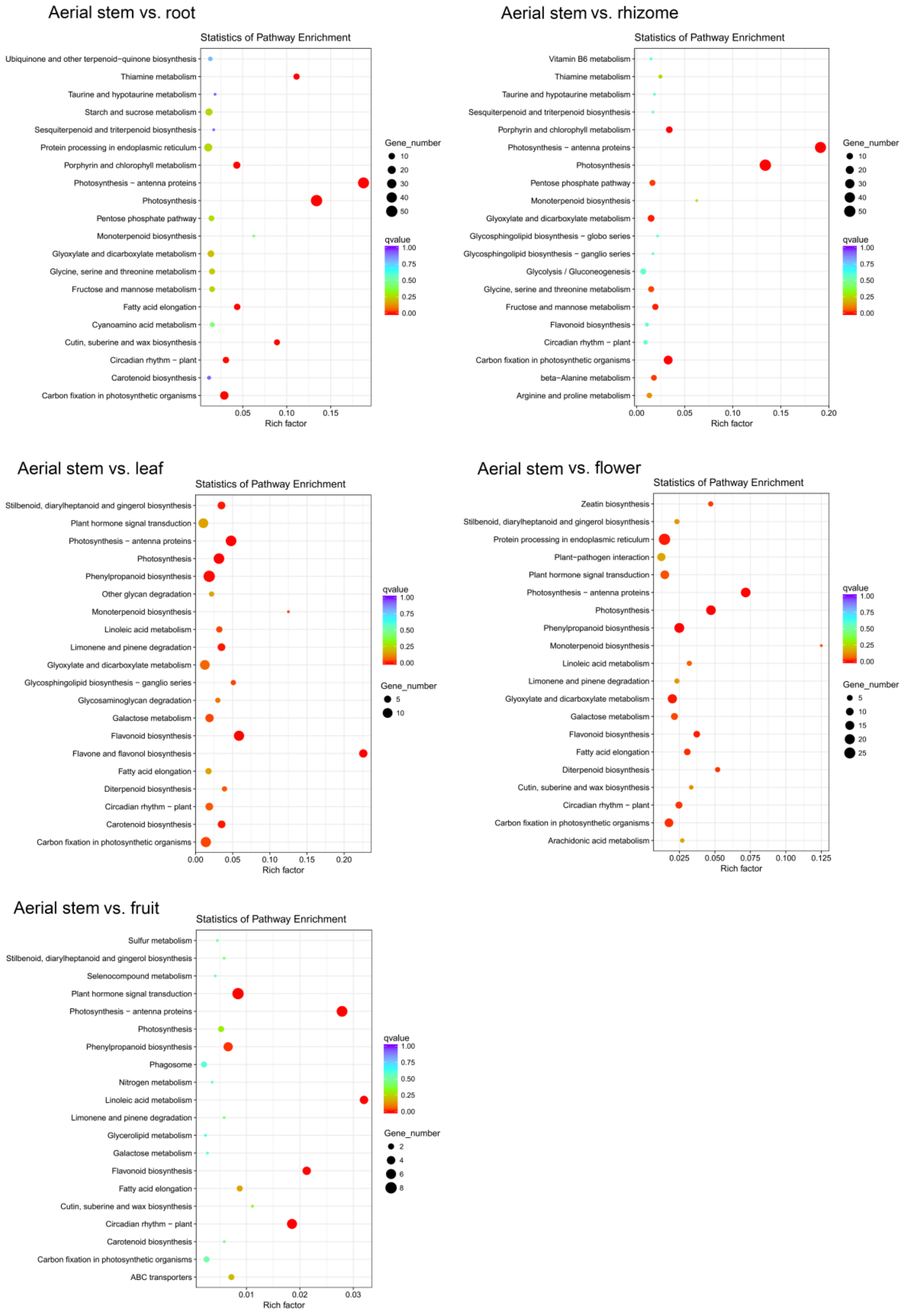

D. Top 20 KEGG enrichment pathways of higher-expressed DEGs in the leaf.

Leaf vs. root

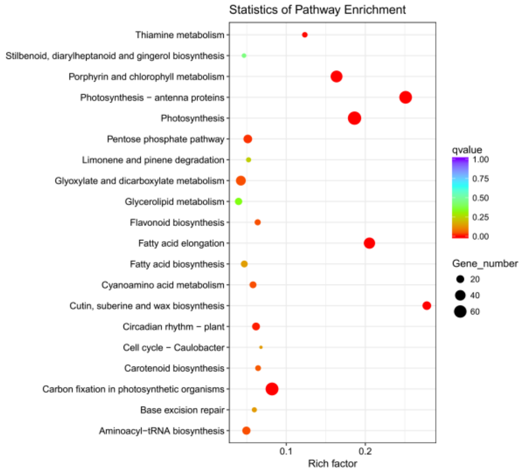

Leaf vs. rhizome

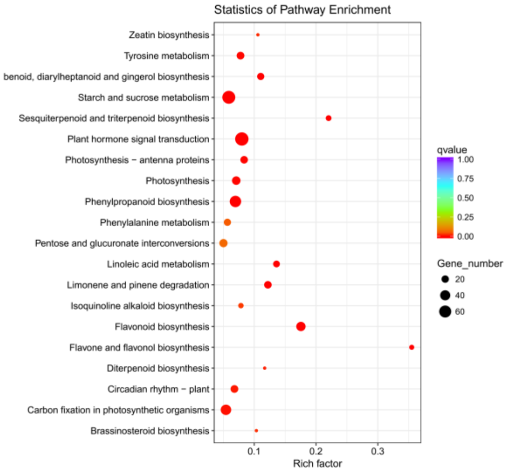

Leaf vs. aerial stem

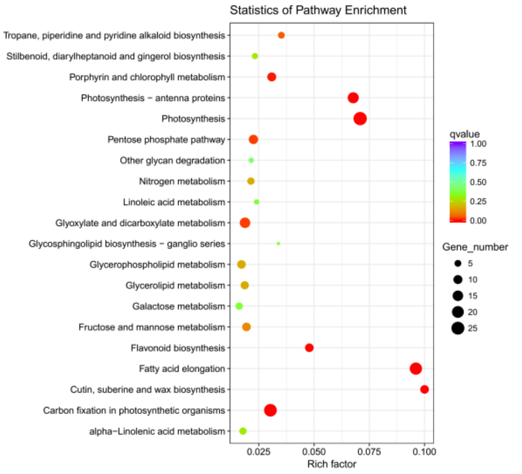

Leaf vs. flower

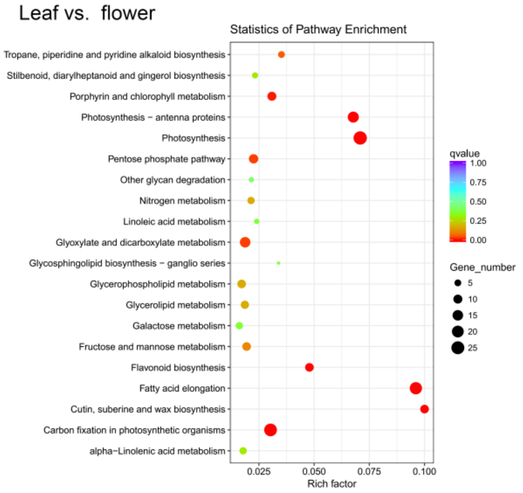

Leaf vs. fruit

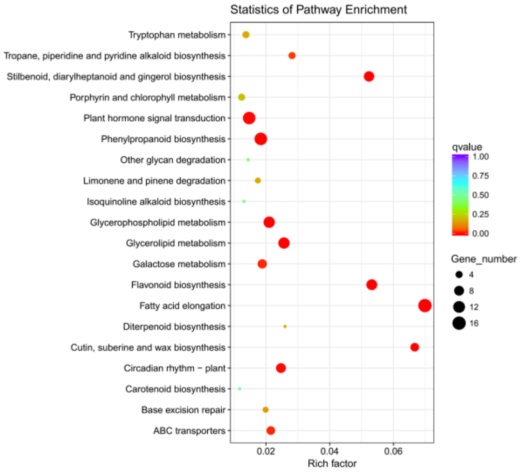

E. Top 20 KEGG enrichment pathways of higher-expressed DEGs in the flower.

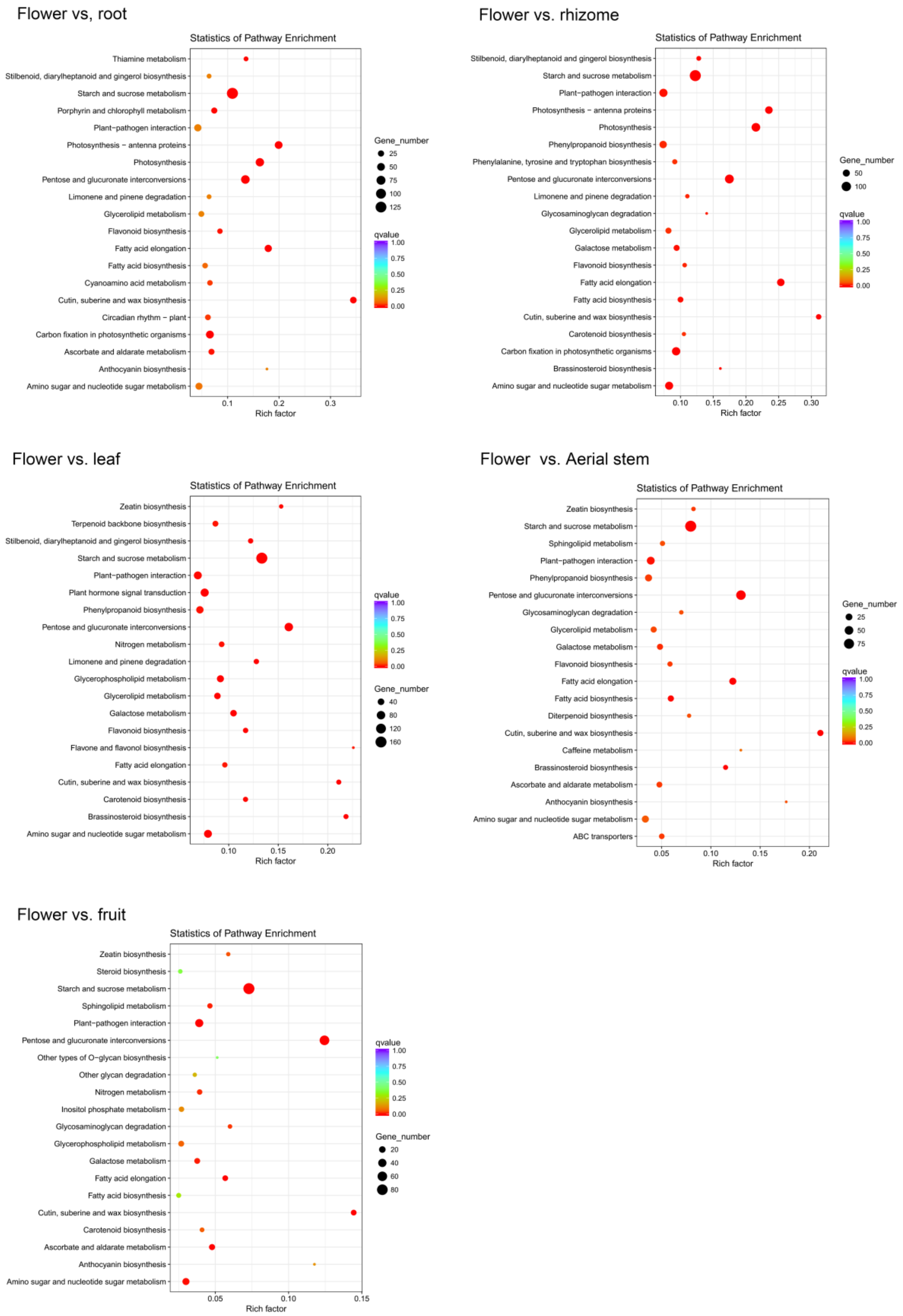

F. Top 20 KEGG enrichment pathways of higher-expressed DEGs in the fruit.

Fruit vs. root

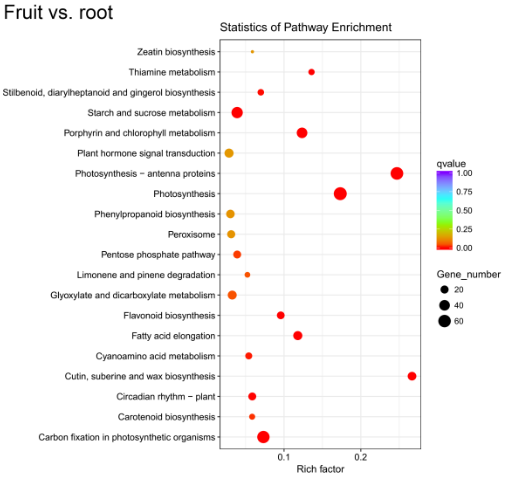

Fruit vs rhizome

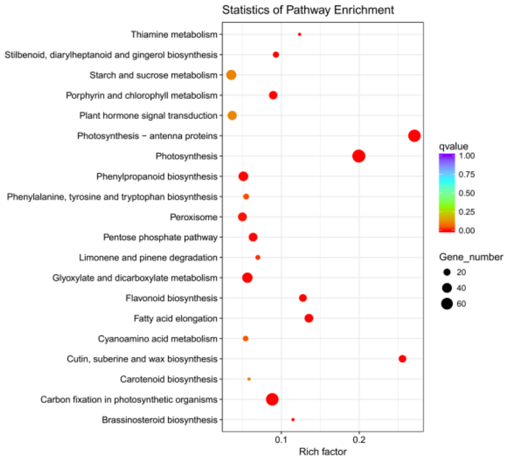

Fruit vs. stem

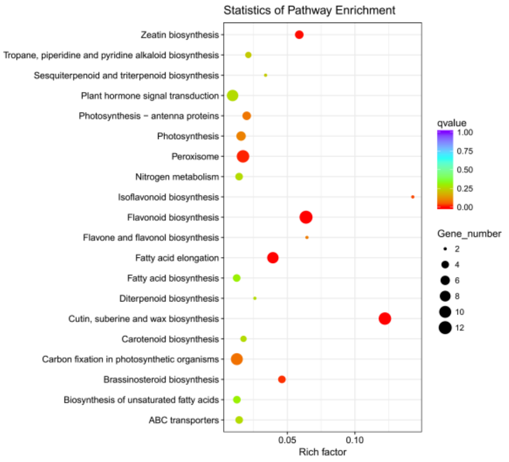

Fruit vs. leaf

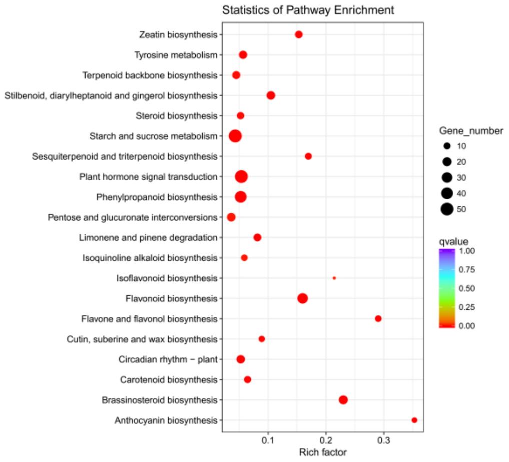

Fruit vs. flower

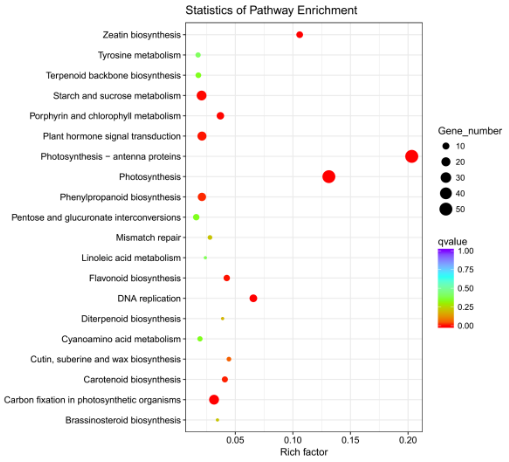

Supplement: Supplementary file 10 [file Image_3.PDF]
